# Comparative Transcriptome Analysis Reveals That Exendin-4 Improves Steatosis in HepG2 Cells by Modulating Signaling Pathways Related to Lipid Metabolism

**DOI:** 10.3390/biomedicines10051020

**Published:** 2022-04-28

**Authors:** Khaoula Errafii, Olfa Khalifa, Neyla S. Al-Akl, Abdelilah Arredouani

**Affiliations:** 1 College of Health and Life Sciences, Hamad Bin Khalifa University, Qatar Foundation, Doha P.O. Box 34110, Qatar; kherrafii@hbku.edu.qa; 2 Diabetes Research Center, Qatar Biomedical Research Institute, Hamad Bin Khalifa University, Qatar Foundation, Doha P.O. Box 34110, Qatar; okhalifa@hbku.edu.qa (O.K.); nalakl@hbku.edu.qa (N.S.A.-A.); 3 African Genome Center, Mohammed VI Polytechnic University (UM6P), Ben Guerir 43151, Morocco

**Keywords:** steatosis, GLP-1R agonist, NAFLD, HepG2, Exendin-4

## Abstract

No therapy exists for non-alcoholic fatty liver disease (NAFLD). However, glucagon-like peptide receptor agonists (GLP-1RAs) showed a beneficial effect on NAFLD, although the underpinning mechanisms remain unclear due to their pleiotropic effects. We examined the implicated signaling pathways using comparative transcriptomics in a cell model of steatosis to overcome pleiotropy. We treated steatotic HepG2 cells with the GLP-1RA Exendin-4 (Ex-4). We compared the transcriptome profiles of untreated steatotic, and Ex-4-treated steatotic cells, and used Ingenuity Pathway Analysis (IPA) to identify the signaling pathways and associated genes involved in the protective effect of Ex-4. Ex-4 treatment significantly reduces steatosis. RNA-seq analysis revealed 209 differentially expressed genes (DEGs) between steatotic and untreated cells, with farnesoid X receptor/retinoid X receptor (FXR/RXR) (*p* = 8.9 × 10^−7^) activation being the top regulated canonical pathway identified by IPA. Furthermore, 1644 DEGs were identified between steatotic cells and Ex-4-treated cells, with liver X receptor/retinoid X receptor (LXR/RXR) (*p* = 2.02 × 10^−7^) and FXR/RXR (*p* = 3.28 × 10^−7^) activation being the two top canonical pathways. The top molecular and cellular functions between untreated and steatotic cells were lipid metabolism, molecular transport, and small molecular biochemistry, while organismal injury and abnormalities, endocrine system disorders, and gastrointestinal disease were the top three molecular and cellular functions between Ex-4-treated and steatotic cells. Genes overlapping steatotic cells and Ex-4-treated cells were associated with several lipid metabolism processes. Unique transcriptomic differences exist between steatotic cells and Ex-4-treated steatotic cells, providing an important resource for understanding the mechanisms that underpin the protective effect of GLP-1RAs on NAFLD and for the identification of novel therapeutic targets for NAFLD.

## 1. Introduction

Non-alcoholic fatty liver disease (NAFLD), a disease marked by an abnormal accumulation of triglycerides in ≥5% of hepatocytes independently of alcohol consumption and other competing liver disorders [1], has become a global health burden, primarily due to the ongoing global rise in sedentary lifestyle and obesity rates [2]. NAFLD ranges from steatosis to non-alcoholic steatohepatitis (NASH), an advanced and more aggressive form of NAFLD, potentially progressing to cirrhosis and hepatocellular carcinoma (HCC). NAFLD affects an estimated 24% of the world’s population [3]; NASH is one of the leading causes of hepatocellular carcinoma and liver transplantation [4]. NAFLD is closely linked to metabolic syndrome [5], insulin resistance (IR) [6], central obesity [7], type 2 diabetes mellitus (T2DM) [8], hypertension [9], and dyslipidemia [10]. NAFLD’s clinical burden is no longer limited to liver-related morbidity and mortality, as the most common causes of death in NAFLD patients are cardiovascular disease (CVD) and cancer, raising worries that NAFLD could be a risk factor for extrahepatic disorders [11]. 

There is presently no approved pharmacotherapy for NAFLD [12]. Diet and exercise-based weight loss is currently the only strategy that has been shown to improve liver function, reduce NAFLD severity, and improve glycemic management and vascular function [13,14,15,16,17,18]. However, achieving the required weight loss, i.e., >5% of body weight, is notoriously difficult, and maintaining it is even more challenging [19].

Recently, NAFLD patients treated with glucagon-like peptide-1 receptor agonists (GLP-1RAs) demonstrated a favorable effect on liver fat content [20,21,22], indicating that these agents could be a novel therapeutic for NAFLD management. 

GLP-1 is a multifunctional hormone produced by the L-cells of the small intestine [23]. Due to the widespread expression of its receptor (GLP-1R), GLP-1 has pleiotropic functions [24]. Among other functions, GLP1 controls glycemia by stimulating glucose-dependent insulin release, decreasing glucagon secretion, potentiating pancreatic β-cell proliferation, and reducing β-cell apoptosis [25]. It also slows gastric emptying [26], and decreases satiety and food consumption by acting on the central nervous system centers [24]. Nevertheless, due to its fast inactivation by the dipeptidyl peptidase 4 (DDP4) [27], the native GLP-1 has a relatively short half-life of 1.5 min [28], making its use as a therapeutic drug impracticable as patients would require a 24-h administration [29]. Because of this impracticality, various longer-acting GLP-1RAs were developed, and some are now licensed for the treatment of T2D, including exenatide (taken twice daily), liraglutide (provided once daily), and the once-weekly agents albiglutide and dulaglutide [30].

Despite the reported beneficial effect of GLP-1RAs in NAFLD [31], the underlying molecular mechanisms remain elusive. On the one hand, some studies attribute the observed improvement to the indirect effect of weight reduction associated with these molecules, ultimately resulting in reduced liver fat content [32]. On the other hand, other studies claim that GLP-1RAs ameliorate NAFLD by directly activating the hepatic GLP-1Rs and the downstream signaling pathways, resulting in the modulation of lipid metabolism pathways and the lowering of hepatic fat content [22,33]. Given the difficulty of weight loss and the maintenance required to improve NAFLD, attempting to discover novel liver fat-reducing medications that do not require weight loss is of critical therapeutic value, not only for obese NAFLD patients but also for normal-weight NAFLD patients, who account for 10 to 15% of all NAFLD cases [34]. Understanding the molecular mechanisms underlying GLP-1Rs’ protective effect is required for this attempt.

We recently reported that the treatment of steatotic human hepatoma HepG2 cells with the GLP-1R agonist Exendin-4 (Ex-4) significantly reduces lipid accumulation and that this effect involves a set of novel long non-coding RNAs [35]. In the present study, we compare the transcriptomic profiles of control, steatotic, and Ex-4-treated steatotic HepG2 cells to examine the differentially expressed mRNAs and signaling pathways, in order to understand the mechanisms behind the improvement of steatosis following Ex-4 treatment and eventually to identify new potential therapeutic targets. We found that Ex-4 treatment up- and down-regulates a significant number of mRNAs associated with relevant metabolic pathways, of which many are related to lipid metabolism, the primary deranged process in NAFLD. 

## 2. Materials and Methods

### 2.1. Preparation of Oleic Acid

The oleic acid (OA) was prepared as in [36] and no diluent was used to dissolve it. In brief, we dissolved the powder OA (O-1008 Sigma-Aldrich, Taufkirchen, Germany) at a final concentration of 12 mM in phosphate-buffered saline (PBS; 137 mM NaCl, 10 mM phosphate, 2.7 mM KCl, and pH 7.4) that contained 11% fatty acid-free bovine serum albumin (FFA-BSA; cat#:0215240110, MP Biomedicals, Santa Ana, CA, USA). The solution was then sonicated and shaken at 37 °C overnight using an OM10 Orbital Shaking Incubator (Ratek Instruments Pty, Ltd., Boronia, Australia). The OA solution was filtered using a 0.22 µm filter, aliquoted, and stored at 4 °C. We used a fresh aliquot for each experiment.

### 2.2. Induction of Steatosis with Oleic Acid

We induced steatosis in HepG2 cells, as we recently reported [35,37]. Briefly, the cells were seeded at a density of 4 × 105 cells/well in 6-well plates until 70% confluence was reached. They were then starved for 6 h in Dulbecco’s Modified Eagle Medium (DMEM) (31966047, Gibco, Waltham, MA, USA) containing 1% fatty-acid free bovine serum albumin (FFA-BSA) instead of 10% fetal bovine serum (10500064, Gibco, Waltham, MA, USA). Upon starvation, steatosis was induced by treating the cells with 400 μM Oleic Acid (OA) for 16hrs in DMEM medium containing 10% BSA. 

### 2.3. Treatment with Ex-4

The following day, the cells were washed and then incubated for three hours in a fresh DMEM solution containing 400 µM OA supplemented or not with 200 nM Ex-4 (E7144-0.1MG, Tocris, Minneapolis, MN, USA). At the end of the experiment, we had three treatment conditions: (1) untreated cells (UCs); (2) steatotic cells (StCs), i.e., cells treated with OA for a total of 19 h; and (3) Ex-4-treated steatotic cells (Ex-4TStCs), i.e., cells treated with OA for 19 h and the DMEM was supplemented with Ex-4 during the last 3 h. All conditions were prepared in triplicate. 

### 2.4. RNA Extraction

We used the Pure Link RNA Mini kit (12183025, Invitrogen, Waltham, MA, USA) to extract the total RNA from UCs, StCs, and Ex-4TStCs, with conditions according to the manufacturer’s instructions. The RNA samples were immediately frozen at −80 °C until use. Before library preparation, we used an RNA broad range assay kit (Q10211, Invitrogen, Carlsbad, CA, USA) and Qubit 2.0 (Thermo Fisher Scientific, Waltham, MA, USA) to measure the RNA concentration. We assessed the RNA quality with an Agilent RNA 6000 Nano Kit (5067-1511, Agilent, CA, USA) and Agilent 2100 Bioanalyzer (Agilent Technologies, Santa Clara, CA, USA) as per the manufacturer’s instructions. 

### 2.5. Library Preparation and RNA Sequencing

The method to prepare the library for RNA sequencing is reported in [35]. Briefly, we used TruSeq RNA Access Library preparation kit (RS-301-2001 and RS-301-2002, Illumina, San Diego, CA, USA) and a starting input material of 100 ng of RNA as directed by the manufacturers. High temperatures and divalent cations were used to fragment the RNA into small pieces, which were immediately reverse-transcribed to first-strand cDNA with random hexamers. The second strand was synthesized by incorporating dUTP instead of dTTP. The sequencing adaptors were ligated to the double-stranded cDNA followed by a single “A” nucleotide adenylation at the 3’ end of blunt fragments. The final library was created by capturing the regions of the transcriptome using sequence-specific probes. The yield of cDNA libraries was quantified using the Qubit dsDNA HS assay kit (Q32855, Invitrogen, Waltham, MA, USA), and the size distribution of the cDNA libraries was determined using the Agilent 2100 Bioanalyzer DNA1000 chip (Agilent Technologies). The clusters were generated on a cBot cluster generation system (Illumina), and sequencing was done on Hiseq 4000 with 150 bp paired-ends. 

### 2.6. Bioinformatics Analysis

We used CLC Genomics Workbench Software Version 21.0.4. (QIAGEN, Hilden, Germany) to look for differences in gene expression between untreated, steatotic, and Ex-4 treated steatotic cells. After retrieving raw RNA sequencing data, pair-end reads were aligned to the Hg38 human reference genome. TPM (Transcript Per Kilobase Million) mapped reads were used to calculate the amount of transcript expression. The ANOVA test was used among the three groups to identify differentially expressed genes (DEGs) with a 2-fold change in absolute value. A statistically significant difference was considered as a *p*-value ≤ 0.05.

### 2.7. Functional and Biological Pathway Analysis

Fold changes in RNA expression of ±2 were applied for filtering, and then the DEGs list was subjected to Ingenuity Pathway Analysis (IPA) (QIAGEN Redwood City, CA, USA) to identify specific networks and pathways. Biological functions and signaling pathways with *p*-values < 0.05 (Benjamini-Hochberg method) were considered significant. The Venn diagrams were created using an online tool, Venny Online tool. 

## 3. Results

### 3.1. Identification of DEGs

**Figure 1:**
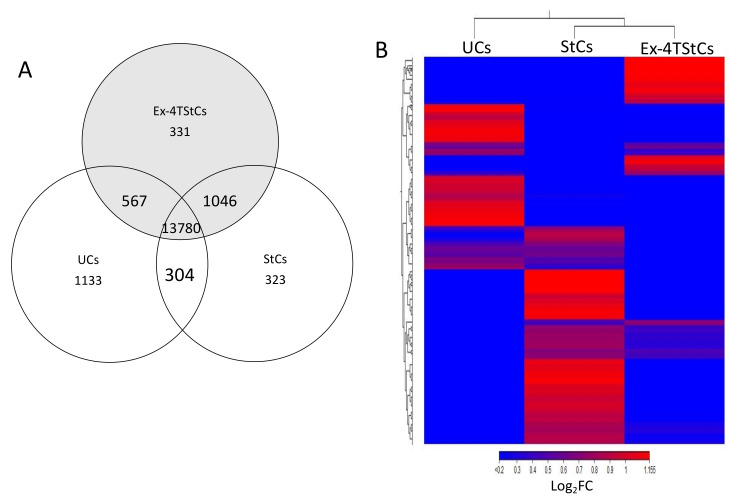
Differentially expressed genes between untreated, steatotic, and Ex-4-treated steatotic cells. (**A**)**.** Venn diagram showing the distribution of the genes affected by steatosis and the treatment with Ex-4 as determined by transcriptomics analysis. (**B**). Log_2_FC heatmap demonstrating overlap in DEG expression patterns in untreated steatotic, and Exendin-4 treated steatotic HepG2 cells. Rows and columns represent genes (1465) and samples, respectively. The heatmap is based on normalized gene expression (GE) values from each dataset. Dendrogram depicts hierarchical clustering of DEGs according to normalized GE values. The blue color indicates downregulated genes while the red indicates the upregulated genes. UCs: untreated cells; StCs: steatotic cells; EX-4-TStCs: Ex-4-treated steatotic cells. *n* = 3 for each condition.

In total, 15,783, 15,452, and 15,723 expressed genes were detected in the UCs, StCs, and EX-4-TStCs, respectively. Of these genes, 13,780 genes were expressed in all three groups; 304, 1046, and 567 genes were identified commonly between each pair of groups (UCs versus StCs, StCs versus EX-4TStCs, and UCs versus EX-4TStCs), while 1133, 323, and 331 genes were discovered exclusively for UCs, StCs, and Ex-4TStCs, respectively (Figure 1A). To identify differentially expressed genes (DEGs) between UCs and StCs and between StCs and Ex-4TStCs, we used CLC Genomics Workbench. Significant differential expression was considered when the fold change is ≥±2 and the *p*-value ≤ 0.05. Thus, a total of 209 and 1644 DEGs were identified when comparing StCs to UCs and Ex-4TStCs to StCs, respectively. The Log_2_ fold change hierarchical clustered heatmap in Figure 1B visualizes the distinct transcriptomic profiles between the different treatment conditions using the top 1465 of the 1853DEGs (this number was used to allow a better visualization). For convenience, the results of each comparison are presented separately. 

### 3.2. Steatotic versus Untreated Cells

**Figure 2:**
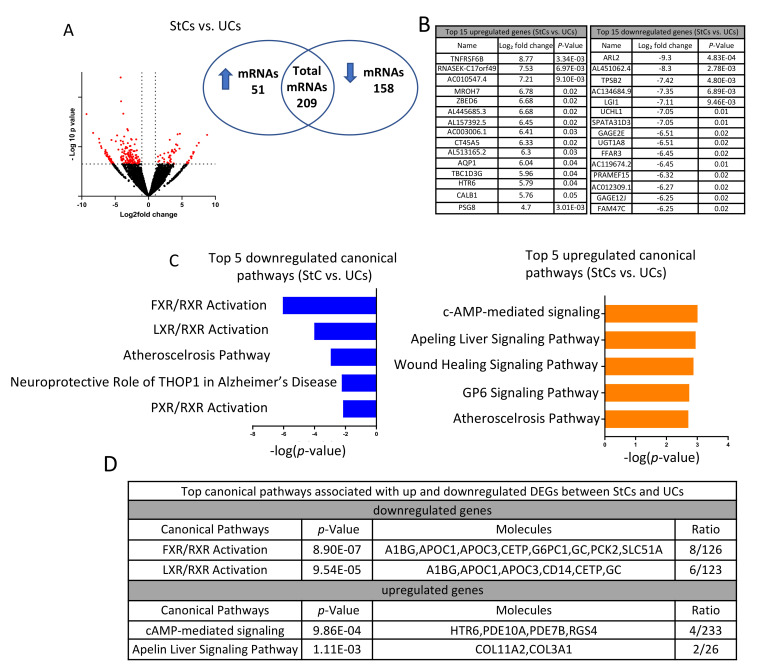
Transcriptome analysis of steatotic versus untreated cells. (**A**) Volcano plot showing that 209 mRNAs were statistically differentially regulated (black dots; ≥2-fold difference, adjusted *p* < 0.05), of which 51 were significantly upregulated and 158 significantly downregulated. (**B**) Top 15 individual up- and downregulated transcripts between StCs and UCs. (**C**) Top five up- and downregulated canonical pathways identified by IPA. (**D**) Top two canonical pathways identified by IPA using up- or downregulated genes and the number of molecules involved. The ratio indicates the number of genes in the analyzed data over the number of genes known to be involved in the pathway.

When we compared the datasets from StCs and UCs, we detected a total of 209 DEGs, with 51 being upregulated and 158 being downregulated (Figure 2A and Table S1). The top 15 up- and downregulated DEGs are listed in the tables shown in Figure 2B. Using the 158 downregulated genes, IPA identified FXR/RXR activation, LXR/RXR activation, atherosclerosis signaling, neuroprotective role of THOP1 in Alzheimer disease, and PXR/RXR activation pathways as the top five significantly downregulated and enriched canonical pathways (Figure 2C, −log (*p*-value) > 1.3). On the other hand, using the 51 upregulated genes, IPA identified cAMP-mediated signaling, apelin liver signaling, wound healing signaling, GP6 signaling, and atherosclerosis signaling pathways as the top five significantly upregulated canonical pathways (Figure 2C; −log (*p*-value) > 1.3). The top two up- and downregulated canonical pathways and the molecules involved in each pathway are shown in the table in Figure 2D. The complete lists of the significantly up- and downregulated canonical pathways are shown in Supplementary Figures S1 and S2.

**Figure 3:**
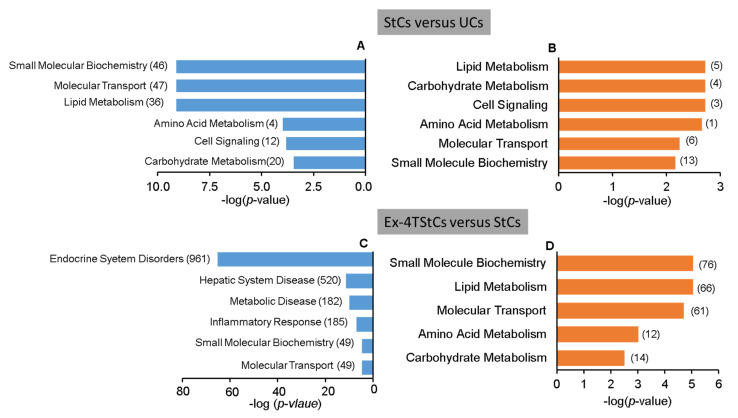
Molecular and cellular functions related to NAFLD (**A**,**B**) Molecular and cellular functions potentially related to NAFLD identified with the down- (**A**) and up- (**B**) regulated DEGs between StCs and UCs datasets. (**C**,**D**) Molecular and cellular functions potentially related to NAFLD identified with the down- (**C**) and up- (**D**) regulated DEGs between StCs and Ex-4-TStCs datasets. The numbers between parentheses indicate the number of genes involved in each function.

Furthermore, IPA identified several molecular and cellular functions when using the 51 upregulated DEGs (Figure S3). Figure 3B shows the biological functions potentially germane to NAFLD, including amino acid metabolism, molecular transport, small molecule biochemistry, lipid metabolism, carbohydrate metabolism, and cell signaling. On the other hand, using the 158 downregulated DEGs, many molecular and cellular functions were discovered (Figure S4). Figure 3A displays the potentially relevant downregulated molecular and cellular activities for NAFLD, including lipid metabolism, molecular transport, small molecule biochemistry, amino acid metabolism, cell signaling, and carbohydrate metabolism.

### 3.3. Steatotic vs. Ex-4-Treated Steatotic Cells

**Figure 4:**
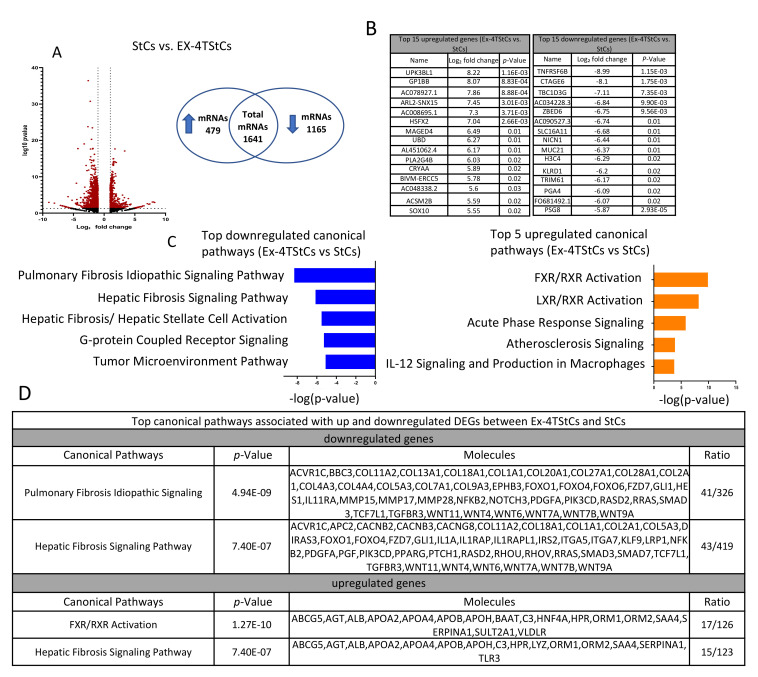
Transcriptome analysis of Ex-4TSC versus SC. (**A**) 1644 mRNAs were determined to be statistically differentially expressed (red dots: >2-fold difference, *p* < 0.05), of which 479 were significantly upregulated and 1165 significantly downregulated. (**B**) Top 15 individual up- and downregulated DEGs between Ex-4TSC and UC. (**C**) Top 5 up- and downregulated canonical pathways identified by IPA. (**D**) Top 2 canonical pathways identified by IPA using up- or downregulated genes and the number of molecules involved. The Venn diagram’s upward and downward arrows indicate upregulation and downregulation, respectively. The ratio indicates the number of genes in the analyzed data over the number of genes known to be involved in the pathway.

A comparison of Ex-4TStCs and StCs datasets identified 1644 DEGs, with 479 being upregulated and 1165 being downregulated (Figure 4A and Table S2). Tables in Figure 4B show the top 15 up- and downregulated DEGs. Using the 479 upregulated genes, IPA identified the FXR/RXR activation, the LXR/RXR activation, the acute phase response signaling, the atherosclerosis signaling, and the IL-12 signaling and production in macrophages as the top five significantly upregulated canonical pathways (Figure 4C). On the other hand, using the 1156 downregulated genes, IPA identified the pulmonary fibrosis idiopathic signaling, the hepatic fibrosis signaling, the hepatic fibrosis/hepatic stellate cell activation, the G-protein coupled receptor signaling, and the tumor microenvironment as the top five significantly downregulated canonical pathways (Figure 4C). The top two up- and downregulated canonical pathways and the molecules involved in each pathway are shown in the table in Figure 4D. The complete lists of the identified significantly up- and downregulated canonical pathways are shown in Figures S5 and S6.

Moreover, IPA identified several molecular and cellular functions when using the 479 upregulated DEGs (Figure S7). Figure 3D shows the upregulated molecular and cellular functions potentially relevant to NAFLD, including lipid metabolism, small molecule biochemistry, amino acid metabolism, carbohydrate metabolism, cell signaling, and molecular transport, as well as the number of molecules involved in each function. Similarly, several molecular and cellular functions were identified using the 1165 downregulated DEGs (Figure S8). Figure 3C depicts the downregulated molecular and cellular processes most likely to be involved in NAFLD, such as endocrine system problems, hepatic system disease, inflammatory response, molecular transport, and small molecule biochemistry, as well as the number of genes associated in each function.

### 3.4. Overlapping Genes between StCs and Ex-4TStCs

**Figure 5:**
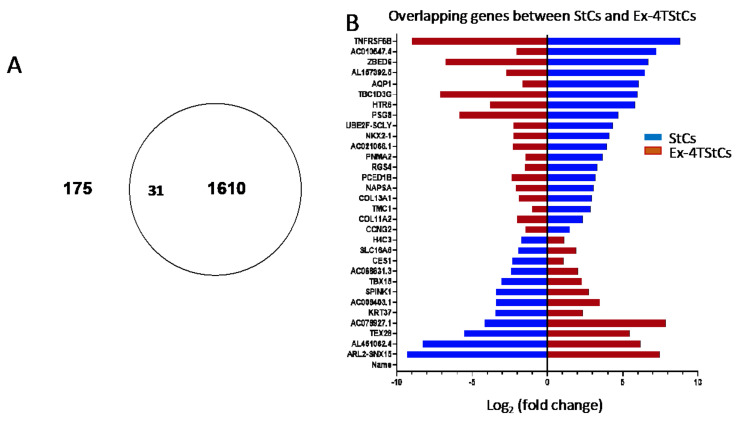
Venn diagram (**A**) and bar graph (**B**) depicting the overlapped DEGs between steatotic cells and Exendin-4 treated steatotic HepG2 cells. The blue color indicates downregulation, while the magenta color indicates upregulation.

To better understand which genes might be functionally related to the protective effects of Ex-4 on OA-induced steatosis, we looked at the list of DEGs that overlap between StCs and EX-4TStCs datasets. As shown in the Venn diagram depicted in Figure 5A, we found 31 overlapping DEGs, of which 19 are downregulated in StCs relative to UCs, but are upregulated after Ex-4 treatment; and 12 are upregulated in StCs relative to UCs, but are downregulated after Ex-4 treatment (Figure 5B and Table S3).

**Figure 6:**
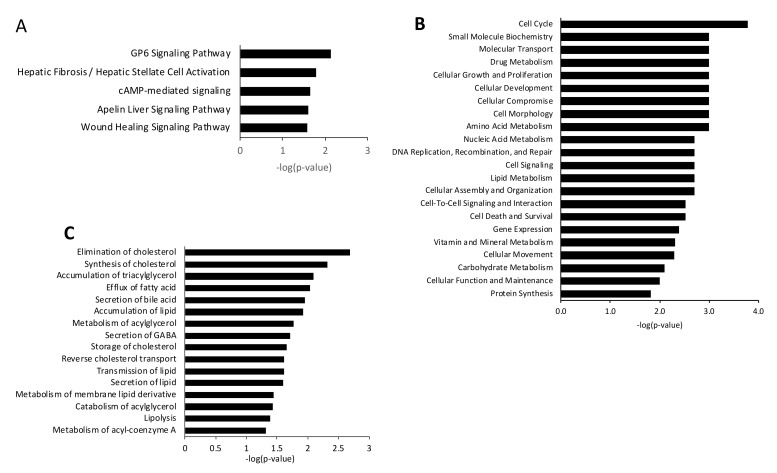
Significant canonical pathways (**A**), cellular and molecular functions (**B**), and lipid metabolism pathways (**C**), enriched with the 31 overlapping genes between StCs and Ex-4TStCs.

Afterwards, we examined the pathway enrichment analysis using the 31 overlapping genes and found that the top five canonical pathways that are significantly enriched include GP6 signaling, hepatic fibrosis/hepatic stellate cell activation, cAMP-mediated signaling, apelin liver signaling, and wound healing signaling (Figure 6A). Additionally, IPA identified several molecular and cellular functions when using the 31 genes (Figure 6B). Several molecular and cellular functions could be potentially relevant to steatosis, including lipid metabolism, small molecule biochemistry, amino acid metabolism, cell signaling, and carbohydrate metabolism. Given that steatosis reflects a disturbance primarily in liver lipid metabolism, we looked into the pathways of lipid metabolism that are significantly enriched with the 31 genes, and identified the elimination of cholesterol, synthesis of cholesterol, accumulation of triacylglycerol, efflux of fatty acid, secretion of bile acid, accumulation of lipids, metabolism of acylglycerol, the transmission of lipids, secretion of lipids, lipolysis, catabolism of acylglycerol, and metabolism of acyl-coenzyme A (Figure 6C).

## 4. Discussion

Because of the world’s rising obesity rates, NAFLD has emerged as a major global healthcare concern. There is currently no approved treatment for NAFLD. However, according to recent research, GLP-1R agonists (GLP-1RAs) may help with NAFLD [38,39]. That said, the mechanisms underpinning the observed NAFLD improvement in response to GLP-1RAs remain elusive because of the pleiotropic effects of these drugs. Indeed, some studies associate GLP-1RAs’ beneficial effect on NAFLD with their weight-loss effect, known to reduce liver fat content [40]. On the other hand, other studies contend that the observed improvement is due to direct stimulation of the hepatic GLP-1R and activation of downstream signaling pathways, notably those linked to lipid metabolism [41,42,43,44]. This idea is supported by the expression of GLP-1R by human hepatocytes [45].

In the present study, we employed comparative transcriptomics to identify the putative signaling pathways and molecules implicated in the protective effect of Ex-4 on steatosis in HepG2 cells. Our rationale for using an in vitro steatosis model is twofold: firstly, as a precursor of NAFLD, hepatic steatosis plays a vital role in the pathological process of NAFLD; and secondly, to overcome the weight-inducing effect of GLP-1RAs in vivo and to investigate the impact of direct stimulation of the hepatic GLP-1R, whose expression by HepG2 cells has already been established [46]. Identifying the signaling pathways and components regulated by direct hepatic GLP-1R stimulation is clinically significant because it may lead to the discovery of novel drug targets and pave the way for the development of medications that can improve NAFLD without requiring weight loss, which is known to be difficult for the majority of obese NAFLD patients [19,47,48]

To the best of our knowledge, this is the first work to look at the signaling pathways regulated in a hepatic cell by directly activating the GLP-1R using a GLP-1R agonist. Our analysis identified key differentially expressed mRNA transcripts and pathways across three cell conditions: (1) untreated (UCs), (2) steatotic (StCs), and (3) steatotic cells treated with Ex-4 (Ex-4TStCs). These findings may help shed important mechanistic light on the protective impact of GLP-1R agonists on NAFLD.

We detected several significant DEGs between UCs and StCs and between StCs and Ex-4TStcS. These DEGs are involved in various critical biological functions and signaling pathways germane to lipid metabolism, the primary deranged process in NAFLD. Indeed, NAFLD arises when the uptake of fatty acids (FA) and triglycerides (TG) from circulation and de novo lipogenesis saturate the rate of FA β-oxidation and very-low-density lipoprotein (VLDL)-TG export [49]. Among the top regulated canonical pathways, the farnesoid X receptor/retinoid X receptor (FXR/RXR) and the liver X receptor/retinoid X receptor (LXR/RXR) activation pathways were downregulated in StCs but upregulated after Ex-4 treatment, indicating that these pathways may play a pivotal role in the Ex-4-induced steatosis improvement that we observe in our model.

The FXR is a major member of the ligand-activated nuclear receptor superfamily [50]. It is a multifunctional receptor that regulates bile acid homeostasis, glucose and lipid metabolism, intestinal bacterial growth, and liver regeneration [51]. Previously, Ma and colleagues (Ma, 2013 #771) demonstrated that treating diet-induced obese mice with the FXR agonist GW4064 reversed hepatic steatosis and reduced plasma lipid levels. Furthermore, activation of FXR inhibits the peroxisome proliferator-activated receptor gamma (PPARγ) expression [52]. PPAR-γ is upregulated in the liver of obese patients with NAFLD, representing an additional reinforcing lipogenic mechanism to sterol regulatory element-binding protein 1c (SREBP-1c) induction in the development of hepatic steatosis [53]. The relevance of PPARγ-regulated hepatic genes and pathways for the development of NAFLD is reported by several studies [54,55]. Interestingly, in an in vitro cell model of steatosis, similar to the one we used in this study, Seo and colleagues [41] recently showed that Ex-4 treatment significantly reduced the expression of both PPARγ and SREBP-1c. We have also observed similar results (paper in press). Furthermore, in diet-induced obese rats, the GLP-1R agonist liraglutide was demonstrated to lower hepatic fat content via decreasing hepatic fatty acid flow via a decrease in PPARγ expression in the liver, implying a restoration of lipid homeostasis [56]. Additionally, the FXR agonist GW4064 reduces the expression of the transmembrane protein CD36 at both protein and mRNA levels [57]. CD36 accelerates the transport of long-chain fatty acids and is overexpressed in diet-induced obesity [58]. The expression of CD36 is also positively correlated with triglyceride (TG) concentration in the liver of NAFLD patients [58]. Besides, overexpression of CD36 increases hepatic uptake of fatty acids in high-fat diet-induced obese mice [59], whereas knockdown of CD36 decreases lipid accumulation in both diet-induced and genetic steatosis [60], suggesting that CD36 is critical in the development of hepatic steatosis. 

As mentioned above, liver de novo lipogenesis is a significant contributor to NAFLD development. FXR-deficient mice exhibited a significant induction of lipogenic genes, such as fatty acid synthase (FAS), SREBP-1c, and stearoyl-CoA desaturase 1 (SCD1) [61]. Consequently, the Ex-4-induced upregulation of the FXR/RXR activation pathway that we observed might be critical for repressing de novo lipogenesis, and thereby reducing steatosis.

In mammalian cells, fatty acid oxidation (FAO), mediated by β-oxidation in mitochondria and peroxisomes and ω-oxidation in cytochromes, plays a significant role in energy generation, especially in periods of low circulating glucose concentrations [62]. Activation of FXR in the liver drives FAO, and although lipid overload and defective mitochondrial β-oxidation characterize NAFLD, defects in ω-oxidation could also contribute to the disease [63]. By inducing PPARα, a transcription factor that is activated by fatty acids and that regulates all three FAO systems, FXR activation increases the expression of FAO genes, including medium-chain acyl-CoA dehydrogenase (MCAD) and long-chain acyl-CoA dehydrogenase (LCAD) enzymes in mitochondria, acyl-CoA oxidase 1 (ACOX1) and enoyl-CoA hydratase (ECH) in peroxisomes, and cytochrome P450, family 4, subfamily a, polypeptide 1 and 3 (CYP4A1 and CYP4A3) in cytochromes [64]. Therefore, by activating the FXR/RXR pathway, Ex-4 might enhance FAO and reduce lipid accumulation in hepatocytes. 

Another prominent canonical pathway downregulated in steatotic cells and upregulated after Ex-4 treatment is the LXR/RXR activation pathway. LXRs are cholesterol sensors that play an essential role in inflammatory control as well as in the regulation of fatty acid, cholesterol, and glucose metabolism [65]. LXRα was overexpressed in the liver of patients with NAFLD or hepatitis C with steatosis [66]. LXRα plays a critical role in fatty acid metabolism in the liver. By inducing SREBP1c, a master regulator of triglycerides and FA synthesis, activation of LXRα promotes the expression of various enzymes involved in fatty acid biosynthesis, including ATP citrate lyase (ACLY), acetyl-CoA carboxylase (ACACA), FAS, SCD1, and glycerol-3-phosphate acyltransferase (GPAT3) [67], and promotes steatosis. Therefore, the upregulation of the LXR/RXR activation pathway we found in response to EX-4 is unlikely to account for the Ex-4-induced steatosis improvement. 

Nevertheless, LXRs exert anti-inflammatory functions in various cells and tissues. For example, Wouters et al. [68] demonstrated that pharmacologic LXRα activation, although it doubles hepatic steatosis, reverses hepatic inflammation in parallel with reversing hepatic cholesterol levels in a high-fat, high-cholesterol-induced NASH mouse model. Moreover, LXR activation also attenuates liposaccharides-induced liver injury in murine NAFLD by inhibiting the pro-inflammatory activity of macrophages [69]. It was also reported that LXR agonist treatment reduces inflammation via the suppression of proinflammatory genes such as cyclooxygenase-2 and inducible nitric oxide synthase [70]. LXR activation also inhibits toll-like receptor (TLR) ligand-dependent inflammatory pathway through ATP-binding cassette 1 (ABCA1) induction [71]. As a result, the beneficial impact of GLP-1R agonist on NAFLD observed in vivo, either in humans or animals, could be attributed partially to the ability of these agents to reduce hepatic inflammation. 

We have found 19 genes that were downregulated in StCs compared to UCs but upregulated after Ex-4 treatment. These 19 genes were enriched in several sub-pathways of lipid metabolism, including lipid droplets, cholesterol homeostasis, lipid catabolic process, cholesterol metabolic process, cholesterol biosynthetic process, positive regulation of cholesterol efflux, cellular response to cholesterol, reverse cholesterol transport, negative regulation of cholesterol storage, and medium-chain fatty acid metabolic process. This observation suggests that the modulation of the expression of these genes by Ex-4 might be crucial for the observed beneficial effect of this drug on steatosis. 

## 5. Conclusions

In the absence of appropriate therapy, NAFLD remains a serious medical condition. GLP-1R agonists have showed potential as NAFLD therapy, although the underlying mechanisms remain unknown. We employed transcriptomics and functional pathway analysis to show that the Ex-4-induced steatosis improvement in HepG2 cells might be partially explained by activation of the FXR/RXR activation pathway via direct stimulation of the GLP-1R. Furthermore, while activation of the LXR/RXR activation pathway by Ex-4 in our model does not reconcile with steatosis reduction in in vivo studies, GLP-1R agonists may employ this pathway to reduce hepatic inflammation and so alleviate NAFLD. Our findings may pave the way for a better understanding of the molecular mechanisms behind the beneficial effect of GLP-1R agonists in NAFLD patients. The main limitation of the present study is the use of the HepG2 cell line instead of primary hepatocytes. Validation of the present results through a comprehensive in vivo investigation of the differentially expressed mRNAs and pathways is warranted in the future.

## Data Availability

All data generated or analyzed during this study are included in this published article (and its Supplementary Information Files) or are available from the corresponding author by reasonable request.

## Supplementary Materials

The following supporting information can be downloaded at: https://www.mdpi.com/article/10.3390/biomedicines10051020/s1, Figure S1: Significant canonical pathways identified by Ingenuity Pathway Analysis (IPA) when using the upregulated DEGs between steatotic and untreated cells; Figure S2: Significant canonical pathways identified by Ingenuity Pathway Analysis (IPA) when using the downregulated DEGs between steatotic and untreated cells; Figure S3: Significant Molecular and Cellular Functions identified by Ingenuity Pathway Analysis (IPA) when using the upregulated DEGs between steatotic and UCs; Figure S4: significant Molecular and Cellular Functions identified by Ingenuity Pathway Analysis (IPA) when using the doenregulated DEGs between steatotic and UCs; Figure S5: Significant canonical pathways identified by Ingenuity Pathway Analysis (IPA) when using the upregulated DEGs between steatotic and Ex-4-treated steatotic cells; Figure S6: Significant canonical pathways identified by Ingenuity Pathway Analysis (IPA) when using the downregulated DEGs between steatotic and Ex-4-treated steatotic cells; Figure S7: Significant Molecular and Cellular Functions identified by Ingenuity Pathway Analysis (IPA) when using the upregulated DEGs between steatotic and Ex-4-treated steatotic cells; Figure S8: Significant Molecular and Cellular Functions identified by Ingenuity Pathway Analysis (IPA) when using the downregulated DEGs between steatotic and Ex-4-treated steatotic cells; Table S1: DEGs between steatotic cells and untreated cells. Upregulated genes are in red; Table S2: DEGs between steatotic cells and Ex-4-treated steatotic cells. Upregulated genes are in red; Table S3: Complete list of 31 overlapping DEGs between steatotic cells and Ex-4-treated steatotic cells. Genes downregulated in steatotic, relative to untreated cells (UCs), and upregulated after Ex-4 treatment are in green, while those upregulated in steatotic cells (StCs), relative to untreated cells, and downregulated after Ex-4 treatment (EX-4-TStCs) are in red.
