# Supplementary material for: Comparative Transcriptome Analysis Reveals That Exendin-4 Improves Steatosis in HepG2 Cells by Modulating Signaling Pathways Related to Lipid Metabolism
Source: Biomedicines. 2022 Apr 28;10(5):1020. doi: 10.3390/biomedicines10051020 (PMC9138370; doi:10.3390/biomedicines10051020)
Supplement: Supplementary file 1 [file biomedicines-10-01020-s001.zip › biomedicines-1647624-supplementary.pdf]

Supplementary data

Comparative transcriptome analysis reveals that Exendin-4 improves steatosis in HepG2 cells by modulating signalling pathways related to lipid metabolism

Khaoula Errafii[1,2, #], Olfa Khalifa[2], Neyla S. AL-AKl[2], Abdelilah Arredouani[1,2*]

[1] College of health and life sciences, Hamad Bin Khalifa University, Qatar Foundation, Doha, Qatar.

[2] Diabetes Research Center, Qatar Biomedical Research Institute, Hamad Bin Khalifa University, Qatar Foundation, Doha, Qatar.

[#] Current address: African Genome Center, Mohammed VI Polytechnic University (UM6P), Lot 660, Hay Moulay Rachid, 43150, Ben Guerir, Morocco

Results

Table S1. DEGs between steatotic cells and untreated cells. Upregulated genes are in red.

Name	Log Fold Change	p-value
ARL2-SNX15	-628.52	4.83E-04
AL451062.4	-315.92	2.78E-03
TPSB2	-171.23	4.80E-03
AC134684.9	-162.66	6.89E-03
LGI1	-138.52	9.46E-03
UCHL1	-132.19	0.01
SPATA31D3	-132.19	0.01
GAGE2E	-91.18	0.02
UGT1A8	-90.93	0.02
FFAR3	-87.46	0.02
AC119674.2	-87.4	0.01
PRAMEF15	-79.67	0.02
AC012309.1	-76.99	0.02
GAGE12J	-76.29	0.02
FAM47C	-75.92	0.02

EVPLL	-72.06	0.02
NPPB	-68.76	0.02
MAGEA9B	-63.46	0.03
WDR87	-60.9	0.03
TREM1	-58.83	0.03
NPR1	-57.85	0.02
MAGEA12	-57.47	0.03
PDE11A_2	-55.34	0.04
ERVV-2	-54.82	0.03
FAM177B	-54.66	0.03
TSPY9P	-53.39	0.04
ZNF723	-49.64	0.04
ZNF681	-49.64	0.04
LEFTY2	-48.42	0.03
GGTLC1	-47.65	0.04
USP17L21	-47.3	5.37E-03
OR2T11	-45.88	0.05
ZNF99	-45.88	0.05
TEX28	-45.88	0.05
AC245748.1	-43.12	0.04
NLGN4X	-39.78	0.04
CHRNA4	-37.23	0.05
AQP10	-29.91	6.06E-03
TFF1	-26.25	3.53E-03
DPP10	-22.97	0.01
TMEM107	-18.14	1.61E-05
AC078927.1	-17.99	5.74E-03
FZD8	-17.55	0.03
LEAP2	-17.4	1.47E-04
SIT1	-17.34	8.38E-03
IL34	-17.28	0.03
GOLGA6L2	-16.93	0.03
GSDMA	-16.53	0.04
DACT2	-16.21	0.02
RNASE2	-15.62	0.03
GGTLC3	-15.54	0.04
SEC14L6	-15.49	0.02
P2RX1	-15.22	0.01
GOLGA6L22	-14.42	6.20E-03
EXOC3L2	-14.17	0.05
LAYN	-14.1	0.03

HAL	-13.75	4.66E-04
LACTBL1	-13.65	0.02
TUBA4B	-13.49	7.48E-03
SLC22A25	-13.39	0.03
IL12RB1	-12.75	0.05
LAIR2	-12.74	0.02
ANGPTL2	-12.52	3.88E-03
AQP8	-12.49	0.02
SLC12A3	-12.22	0.02
PSTPIP1	-11.29	0.02
VNN1	-11.08	0.02
KRT37	-11.08	0.01
GUCY2C	-10.86	0.04
AC008403.1	-10.64	0.04
SPINK1	-10.55	0.01
INHA	-10.21	0.01
G6PC	-10.06	0.03
NRL	-9.6	0.01
GC	-9.59	0.05
MATK	-9.34	0.05
SLC28A1	-9.26	0.02
ADGRD1	-9.23	0.01
SLC25A47	-9.17	0.03
KRTAP5-7	-9.13	0.03
MUC5B	-9.11	1.94E-03
GMFG	-8.85	0.03
ALPL	-8.62	7.16E-03
NXPH3	-8.54	0.02
GPX3	-8.48	2.22E-03
ATOH8	-8.36	0.04
SYN3	-8.31	0.04
CD14	-8.28	0.05
TBX15	-8.26	0.02
SYP	-8.21	0.02
SPN	-8.11	0.02
BIRC7	-7.86	0.02
CETP	-7.47	0.04
PERM1	-7.43	0.04
TNNI2	-7.26	0.01
KLHDC7A	-7.22	0.05
SLC11A1	-7.16	0.03

FXYD1	-6.96	9.48E-03
ENG	-6.81	0.02
AC013489.1	-6.8	0.03
SLC51A	-6.79	0.04
ANGPTL8	-6.78	0.02
GPD1	-6.7	0.02
CPZ	-6.66	0.04
CLDN6	-6.57	0.02
VAMP5	-6.56	9.37E-03
S100P	-6.41	0.02
PLA2G2A	-6.37	0.01
UNC93A	-6.17	0.02
SERPINE1	-6.15	4.89E-03
SHBG	-5.9	0.04
BPIFB2	-5.82	0.03
TNS1	-5.77	5.21E-03
IGF2	-5.76	0.01
LY6D	-5.75	0.05
LCN15	-5.74	0.03
APOC3	-5.67	0.04
APOC1	-5.6	0.04
A1BG	-5.43	0.02
ITGB2	-5.42	0.04
HGFAC	-5.41	0.05
HPN	-5.35	0.04
AC068631.3	-5.29	0.04
PDE4C	-5.29	0.03
RASD1	-5.28	0.04
CES1	-5.11	0.05
SPAG4	-5.02	0.03
CREB3L3	-4.98	0.02
SLC1A2	-4.79	0.04
LOXL4	-4.76	0.02
LOX	-4.69	0.03
MASP1	-4.59	0.04
NAV2	-4.27	0.02
ROMO1	-4.25	8.02E-03
TMPRSS6	-4.24	0.04
CRYAA2	-4.07	0.05
TRPV2	-4.01	0.05
SELENOP	-3.98	0.05

TIMP3	-3.93	0.03
SLC16A6	-3.85	0.02
SEMA3G	-3.66	0.05
RPS21	-3.61	5.06E-03
PLCXD1	-3.58	0.02
PLIN2	-3.56	0.03
AP001931.2	-3.52	0.03
ALDH4A1	-3.43	0.03
GDF15	-3.41	0.01
H4C3	-3.35	0.01
SLC25A10	-3.27	0.05
INSIG2	-3.17	0.05
EFNA1	-3.16	0.04
KHK	-3.05	0.04
TUBB2B	-2.97	0.03
PCK2	-2.91	0.04
CTSD	-2.67	0.05
SLC5A6	-2.66	0.05
RHOB	-2.61	0.03
ABCD1	-2.49	0.04
**CD109**	**2.66**	**0.02**
**CCNG2**	**2.75**	**0.04**
**ANXA3**	**3.08**	**0.04**
**GDA**	**3.38**	**0.02**
**AP1S3**	**4.16**	**0.05**
**AREG**	**4.27**	**0.03**
**EMP1**	**5.04**	**0.03**
**COL11A2**	**5.05**	**0.04**
**IL18**	**5.13**	**0.02**
**TMC1**	**7.12**	**0.04**
**COL13A1**	**7.69**	**0.02**
**NAPSA**	**8.16**	**0.05**
**SOGA3**	**8.2**	**0.03**
**LHX5**	**8.2**	**0.05**
**LY6K**	**8.65**	**0.05**
**COL3A1**	**8.91**	**0.03**
**INSIG1**	**8.99**	**5.28E-03**
**PCED1B**	**9**	**0.02**
**NXF3**	**9.25**	**0.04**
**TPPP3**	**9.78**	**0.02**
**RGS4**	**9.94**	**0.03**

RBM46	10.04	0.03
LHX1	10.09	0.04
PDE7B	11.13	0.02
DCLK1	11.45	0.01
PNMA2	12.41	0.02
FXYD7	13.48	0.05
NME9	13.72	0.05
PDE10A	14.01	0.02
CHST2	14.57	0.02
AC021066.1	15.23	0.01
UTS2	17.1	0.01
NKX2-1	17.32	8.05E-03
LBH	19.88	8.78E-03
UBE2F-SCLY	20.41	3.18E-03
GAB3	24.71	0.01
PSG8	25.94	3.01E-03
CALB1	54.04	0.05
HTR6	55.24	0.04
TBC1D3G	62.46	0.04
AQP1	65.97	0.04
AL513165.2	78.91	0.03
CT45A5	80.67	0.02
AC003006.1	85.15	0.03
AL157392.5	87.6	0.02
AL445685.3	102.67	0.02
ZBED6	102.73	0.02
MROH7	109.93	0.02
AC010547.4	148.31	9.10E-03
RNASEK-C17orf49	185.12	6.97E-03
TNFRSF6B	437.85	3.34E-03

Table S2. DEGs between steatotic cells and Ex-4-treated steatotic cells. Upregulated genes are in red.

Name	Log Fold Change	p-value
TNFRSF6B	-508.49	1.15E-03
CTAGE6	-273.72	1.75E-03
TBC1D3G	-137.85	7.35E-03
AC034228.3	-114.56	9.90E-03
ZBED6	-107.46	9.56E-03
AC090527.3	-106.8	0.01
SLC16A11	-102.57	0.01
NICN1	-86.95	0.01
MUC21	-82.56	0.01
H3C4	-78.47	0.02
KLRD1	-73.72	0.02
TRIM61	-71.92	0.02
PGA4	-68.05	0.02
FO681492.1	-67.1	0.02
PSG8	-58.58	2.93E-05
SPTY2D1OS	-56.48	0.03
RIC3	-56.09	0.03
CGB8	-52.64	0.03
TLR9	-48.8	0.04
CHAT	-48.8	0.04
LIM2	-44.98	0.04
CELF3	-42.86	0.04
CYP7A1	-41.18	0.04
MUC19	-41.18	0.04
CLC	-41.18	0.04
C2orf66	-40.63	0.04
PLEKHS1	-40.54	0.05
CITED4	-38.44	0.04
AL512506.3	-38.42	0.05
RBPJL	-38.42	0.05
AC011498.4	-37.6	0.05
LDLRAD2	-37.31	2.87E-04
HAPLN2	-36.24	0.05
BDKRB1	-36.24	0.05
OLFML1	-36.23	0.05
SLC12A5	-36.23	0.05
GGT1_2	-25.18	1.88E-03
AL662899.3	-23.06	3.92E-03

CDC42EP2	-22.8	5.95E-09
PRRT2	-21.74	1.72E-04
DNAH8	-21.25	2.01E-03
TPSG1	-20.97	5.18E-03
GNB4	-20.7	2.19E-03
AC037459.1	-20.45	1.17E-03
AC010422.3	-20.14	2.76E-03
KYAT1	-19.15	1.80E-18
RASL10A	-17.45	6.63E-03
ARID3C	-16.27	6.42E-03
HTR6	-14.3	1.42E-03
IGSF23	-14.06	8.37E-03
CHAD	-13.94	5.37E-05
RSPO1	-13.17	0.01
BCL2L14	-12.84	0.02
TNFAIP8L2	-12.7	0.02
EMILIN3	-12.7	0.02
DLGAP3	-12.41	0.01
NLRP6	-11.45	0.02
CTF1	-11.42	9.23E-04
STAB1	-10.99	0.03
C19orf67	-10.99	0.03
TXLNB	-10.75	0.02
DEPP1	-10.72	1.68E-04
LRRC32	-10.45	3.41E-04
PVRIG	-10.16	0.03
ANGPTL6	-9.93	6.95E-03
KLK12	-9.83	1.71E-03
TBATA	-9.66	0.04
OR2H2	-9.54	9.87E-04
NOG	-9.54	4.29E-06
PPFIA4	-9.45	9.49E-20
AC138696.1	-9.3	6.99E-03
LHX9	-9.21	0.04
SLC14A2	-9.17	2.33E-03
GPR21	-9.08	0.03
SLC4A1	-9.08	0.03
USP17L17	-9.03	5.60E-05
HLA-DQA2	-9.02	0.01
NUTM2F	-8.98	4.21E-05
SH2D3C	-8.81	7.22E-08

FAM243B	-8.75	0.02
GPR171	-8.67	0.01
PRICKLE4	-8.56	6.80E-05
ROPN1	-8.53	0.04
CCL27	-8.4	0.02
FKBP1C	-7.98	0.04
BLK	-7.98	0.04
ROBO4	-7.97	0.05
SLC39A2	-7.97	0.05
KISS1	-7.82	1.05E-03
TIAF1	-7.73	0.02
NYAP1	-7.63	7.79E-14
MAMSTR	-7.62	6.07E-03
SRPK3	-7.58	2.99E-03
KLK1	-7.57	2.92E-03
ATP5MGL	-7.54	8.96E-03
KIRREL2	-7.48	4.42E-05
LRRC73	-7.32	1.06E-06
CCL3L3	-7.09	0.03
AL117348.2	-7.07	0.01
CHRND	-7.06	2.57E-04
SLITRK6	-7.03	0.01
AP001781.2	-6.99	0.04
CSRNP3	-6.95	3.82E-05
GPR55	-6.95	0.01
RNF224	-6.95	0.01
AC010463.1	-6.95	1.17E-03
DUSP10	-6.88	2.00E-12
VSIG8	-6.81	9.46E-03
SLC22A13	-6.79	7.54E-03
PTX3	-6.77	0.03
PHOSPHO1	-6.76	3.10E-03
FGF17	-6.73	0.01
FRZB	-6.68	4.38E-04
AL157392.5	-6.68	1.48E-03
ENTPD8	-6.64	3.74E-03
AC068896.3	-6.59	9.97E-03
DAPP1	-6.58	0.03
PAGE2B	-6.55	9.83E-05
PABPN1L	-6.48	0.02
SPEF1	-6.47	0.02

CPNE5	-6.46	0.03
SNAI3	-6.42	5.67E-05
LILRA6	-6.42	3.72E-03
C1orf162	-6.36	6.21E-03
CGB1	-6.33	0.01
LDLRAD1	-6.3	0.01
SEC14L5	-6.24	5.81E-03
RAPGEF4	-6.21	2.68E-06
GAS1	-6.21	2.24E-03
TMEM74B	-6.21	2.07E-03
PSG6	-6.2	0.01
RBM20	-6.16	1.75E-24
SCARA5	-6.14	0.04
ANKRD2	-6.14	0.04
HSFX3	-6.14	0.04
STEAP4	-6.08	4.26E-03
MARCHF10	-6.07	0.02
CHODL	-6.07	0.02
PON1	-6.06	0.02
LAG3	-6.05	1.08E-04
MAP3K8	-6.04	6.35E-07
LRMP	-6.01	0.05
CHST1	-5.99	6.14E-04
RNF152	-5.99	4.20E-03
IL7	-5.98	0.05
GRB7	-5.98	3.82E-37
AC107959.5	-5.95	0.03
SIX2	-5.88	1.40E-03
GAL3ST1	-5.87	2.13E-05
TDGF1	-5.83	0.04
GFAP	-5.8	0.02
ATP10A	-5.79	0.05
TSLP	-5.77	3.92E-03
CRHR1	-5.77	7.38E-03
C7orf61	-5.73	4.15E-03
FOXL1	-5.72	1.46E-12
CLIC5	-5.7	8.14E-03
ADRB1	-5.69	1.54E-04
MT-ATP8	-5.62	1.91E-05
ACVR1C	-5.61	9.63E-05
PSG3	-5.61	0.01

GMNC	-5.55	6.47E-03
SPRR2D	-5.54	0.01
AP003419.1	-5.51	0.05
GPR20	-5.5	3.40E-04
GRIK2	-5.48	7.22E-03
TEN1-CDK3	-5.45	0.01
SOWAHB	-5.44	1.64E-06
KLF9	-5.44	3.38E-06
CHRNA2	-5.41	0.02
USP17L13	-5.4	6.30E-03
DLL4	-5.39	3.78E-06
SNAP25	-5.33	3.36E-03
COL5A3	-5.3	0.02
CARNS1	-5.24	6.60E-03
GPRIN3	-5.18	4.00E-05
COL2A1	-5.18	0.02
AC005726.1	-5.15	6.55E-03
GUCY2F	-5.15	0.04
PCED1B	-5.14	0.02
BRICD5	-5.12	5.28E-03
GPR37L1	-5.11	0.05
ASIC4	-5.08	4.29E-05
CXCR4	-5.05	3.45E-07
CLEC3B	-5.05	8.81E-03
FAM205A	-5.03	4.76E-03
SOX30	-5.02	3.94E-03
C8orf88	-4.96	0.04
CASTOR1	-4.96	0.02
AC098582.1	-4.91	6.63E-08
AC021066.1	-4.91	5.00E-06
GPR182	-4.9	0.02
UBE2F-SCLY	-4.88	3.42E-04
CCDC8	-4.87	0.04
CRYBB3	-4.87	0.02
CASZ1	-4.85	2.00E-15
NKX2-1	-4.85	2.05E-06
ATP13A4	-4.84	0.02
ELF3	-4.77	1.77E-31
AC097634.4	-4.77	9.44E-03
AC074143.1	-4.77	1.78E-04
SLC4A9	-4.76	0.02

COL20A1	-4.76	0.02
WNT4	-4.75	1.58E-09
WNT6	-4.75	4.76E-04
AC055839.2	-4.73	2.22E-03
MT-ND1	-4.72	2.29E-05
WNT7A	-4.67	0.04
CARD11	-4.67	0.01
ERFL	-4.67	0.03
PSD2	-4.66	2.65E-04
ZSWIM4	-4.66	2.34E-18
BMF	-4.64	2.94E-05
TRIM17	-4.62	3.85E-03
KIF7	-4.61	3.20E-03
CREG2	-4.59	0.02
MT-CYB	-4.59	7.06E-09
PCDHGC3	-4.58	7.24E-04
MAMDC4	-4.58	2.96E-06
AL603832.3	-4.57	1.33E-05
NR5A2	-4.57	2.25E-04
OTUD1	-4.57	4.82E-10
IL1RAP	-4.53	5.73E-08
ICAM5	-4.53	3.30E-16
SLC2A12	-4.51	4.78E-09
GNG14	-4.5	0.04
FBLN2	-4.47	0.03
CXXC4	-4.46	6.99E-03
RHBDL1	-4.46	3.87E-04
OR7D2	-4.46	6.63E-03
CAPN12	-4.46	1.16E-03
PRR15	-4.45	9.19E-03
COL7A1	-4.44	2.16E-08
PLSCR4	-4.44	4.39E-07
DHRS3	-4.43	1.31E-08
SPEG	-4.43	2.46E-11
CPXM1	-4.39	0.01
PAGE2	-4.39	0.03
HPCA	-4.36	0.02
SOX8	-4.36	2.57E-04
NKX6-1	-4.35	1.94E-09
HSPB6	-4.35	0.02
IL1A	-4.33	0.03

NAPSA	-4.33	1.38E-03
SNORC	-4.32	0.01
MSLNL	-4.3	0.02
GIPR	-4.3	2.80E-07
ABCG4	-4.29	9.43E-06
FOXH1	-4.27	1.36E-03
NECTIN4	-4.26	1.77E-10
IL31RA	-4.26	3.18E-04
ZNF503	-4.25	1.29E-14
ARSJ	-4.24	1.94E-03
PLAG1	-4.24	0.03
MT-ND6	-4.24	2.66E-06
HLX	-4.23	5.48E-03
RUFY4	-4.23	0.03
KCNIP3	-4.22	1.10E-03
THEGL	-4.21	0.04
KCTD19	-4.21	0.04
SLC45A3	-4.2	3.69E-08
IL10RA	-4.2	1.79E-08
AL451136.1	-4.19	0.02
COL14A1	-4.19	0.02
AC010547.4	-4.19	8.07E-03
RBM24	-4.18	7.94E-06
SPNS2	-4.18	3.14E-09
DACT1	-4.17	2.02E-05
TAFA3	-4.16	0.04
CGB7	-4.16	4.06E-06
DLL1	-4.15	1.15E-13
EBF3	-4.15	3.89E-05
TMEM229B	-4.15	2.05E-03
TMEM169	-4.14	1.54E-03
PKNOX2	-4.14	0.04
TCP10L	-4.14	0.01
BBC3	-4.13	3.47E-05
PTCH1	-4.11	5.08E-21
ARHGEF18	-4.11	1.84E-08
TIAM2	-4.09	4.29E-13
SMTNL2	-4.09	5.55E-06
BOC	-4.08	4.66E-06
CNGA1	-4.08	7.78E-04
CYP26A1	-4.07	0.03

PPFIA2	-4.07	0.03
ZBTB18	-4.06	7.76E-07
COL11A2	-4.06	3.98E-07
FAM214B	-4.06	3.88E-09
KCNN1	-4.06	1.94E-03
HCAR2	-4.04	0.02
KCNB1	-4.04	3.70E-04
GAB1	-4.03	3.11E-15
FAM209A	-4.03	0.02
SHISA8	-4.03	0.02
ARTN	-4.02	4.22E-03
CDKN1C	-4.02	7.12E-09
KCTD11	-4.02	4.01E-04
PEAR1	-4.01	5.58E-08
GPSM3	-4.01	0.04
LMO2	-4.01	1.60E-03
BIRC3	-4.01	6.68E-06
NOL4L	-4.01	2.93E-11
RBMS3	-4	0.05
PIWIL2	-4	0.03
PCDHA1	-3.96	0.04
RBBP8NL	-3.96	6.22E-04
CCDC110	-3.94	1.97E-08
LDLRAD4	-3.92	6.54E-03
AP001458.2	-3.87	0.05
PLEKHG4B	-3.86	0.03
CFAP99	-3.85	1.04E-03
TAC3	-3.85	0.04
AC090517.4	-3.85	0.01
SPON1	-3.84	0.03
WDR97	-3.83	6.56E-03
RASD2	-3.82	1.27E-05
PTPN7	-3.81	0.02
TMED7-TICAM2	-3.81	1.76E-04
FRS3	-3.8	9.63E-08
PRSS22	-3.79	1.22E-08
TAPBPL	-3.78	0.02
HSFX1	-3.78	1.32E-04
DIPK1A	-3.76	5.67E-07
ZNF439	-3.75	0.03
DNASE2B	-3.74	0.02

TMEM61	-3.73	8.28E-05
PNRC1	-3.73	7.35E-14
COL13A1	-3.73	3.84E-11
ENO2	-3.73	3.43E-24
AMIGO2	-3.73	8.71E-03
CAPS	-3.73	2.36E-03
PIGZ	-3.72	2.75E-03
AC004223.3	-3.72	0.01
CD72	-3.71	3.54E-03
TMEM88	-3.7	0.04
PLCXD2	-3.69	1.68E-06
SH3D21	-3.68	1.91E-08
EPHB3	-3.68	5.17E-10
CA3	-3.68	0.05
KCNMB3	-3.67	0.05
ALDH8A1	-3.67	4.34E-03
AOAH	-3.67	0.03
TBC1D26	-3.67	0.02
PPM1K	-3.66	1.14E-05
AL162231.3	-3.66	0.01
HSF4	-3.66	2.56E-06
HIF3A	-3.66	0.05
CECR2	-3.66	2.23E-18
ARSA	-3.65	1.12E-06
CNTNAP3C	-3.64	9.16E-07
ANKRD33B	-3.62	2.15E-06
COL27A1	-3.62	2.13E-07
BHLHE41	-3.62	7.90E-03
TMCC3	-3.62	2.51E-06
SLC2A4	-3.62	2.65E-07
LMTK3	-3.61	3.17E-08
MYCL	-3.6	1.48E-06
ADGRD2	-3.6	0.05
KLF15	-3.59	9.61E-03
GEM	-3.58	7.95E-14
SCEL	-3.58	0.01
TEX29	-3.58	0.03
CITED1	-3.58	3.07E-03
TPPP	-3.57	1.29E-04
GAL3ST4	-3.57	1.96E-04
PITX3	-3.57	3.08E-04

NCKAP5L	-3.57	6.78E-09
SERTAD3	-3.57	2.43E-04
CCDC154	-3.56	0.04
HCFC1R1	-3.56	1.15E-07
ZNF703	-3.55	1.15E-04
IL2RB	-3.55	0.04
CILP2	-3.53	4.26E-03
GLI1	-3.52	1.72E-05
PTPRH	-3.51	1.62E-18
SSC5D	-3.51	2.72E-05
GOLGA6B	-3.49	0.03
YPEL2	-3.49	8.09E-04
RAPGEF3	-3.48	1.17E-03
DOCK8	-3.47	0.02
NOXA1	-3.47	4.34E-04
KRTAP5-1	-3.47	6.86E-03
POU2F2	-3.47	0.03
MT-ATP6	-3.47	8.75E-07
EFEMP2	-3.46	8.55E-05
AC137834.1	-3.46	2.87E-05
ARID3A	-3.46	7.36E-09
TCAF2C	-3.45	4.08E-08
EYA1	-3.45	5.47E-04
ZCCHC24	-3.45	6.30E-03
TMEM92	-3.45	1.66E-05
COL1A1	-3.44	3.56E-08
ST3GAL5	-3.43	3.88E-06
FLRT3	-3.42	5.88E-03
PLXNB3	-3.42	6.52E-03
C11orf42	-3.41	0.04
MYT1	-3.41	7.99E-13
GRAP	-3.4	0.04
PRR15L	-3.4	0.04
Z82190.2	-3.38	0.05
PCOLCE	-3.37	5.97E-06
ZNF467	-3.37	5.21E-05
ANKRD34A	-3.36	0.03
PDGFA	-3.36	2.48E-05
ARHGEF40	-3.36	2.56E-09
MT-CO1	-3.36	4.06E-06
TG	-3.35	0.02

NFATC4	-3.35	1.01E-05
INSIG2	-3.34	3.29E-08
DBP	-3.34	8.39E-08
SPATA31A3	-3.33	4.84E-03
AC073610.2	-3.33	1.13E-05
PDE3A	-3.32	5.83E-03
APOBEC3D	-3.32	1.78E-04
SLC45A1	-3.31	7.12E-13
THPO	-3.31	0.04
BCL6	-3.31	4.47E-15
IRF2	-3.31	5.94E-09
MGAM	-3.31	1.70E-05
SLIT1	-3.31	1.97E-03
AC004997.1	-3.31	1.39E-03
NEURL1B	-3.3	6.73E-03
GSDMB	-3.3	9.28E-11
MISP3	-3.3	5.73E-06
SPATA31A1	-3.29	4.98E-03
EVA1B	-3.28	2.62E-04
SOX2	-3.28	0.04
AGER	-3.28	2.24E-05
BCL3	-3.28	3.09E-08
TESK2	-3.26	2.97E-07
TMCC2	-3.26	6.83E-07
MT-ND4L	-3.26	1.02E-03
LRRC24	-3.25	0.01
SYNE3	-3.25	3.35E-06
STON1-GTF2A1L	-3.24	2.50E-03
STX1A	-3.24	1.60E-10
SEMA3C	-3.24	1.82E-04
DLX4	-3.24	2.86E-06
ENGASE	-3.24	8.61E-07
ANKZF1	-3.23	2.53E-15
NDUFC2-KCTD14	-3.23	5.60E-03
AQP1	-3.22	0.03
USP17L7	-3.22	1.53E-03
CYP2E1	-3.22	8.27E-03
WNT11	-3.22	0.04
PGF	-3.22	2.86E-03
MT-ND4	-3.21	9.47E-07
SPRED3	-3.2	0.02

CA8	-3.19	5.27E-04
HCN3	-3.18	1.39E-04
NOVA2	-3.18	3.08E-07
L3MBTL1	-3.18	4.15E-06
GPR153	-3.17	3.25E-04
GRIP2	-3.17	0.02
RHPN1	-3.17	2.02E-07
AC008878.1	-3.17	5.80E-04
CTSK	-3.16	4.99E-05
SLCO5A1	-3.16	1.74E-04
TCTE1	-3.15	0.04
GPR146	-3.15	8.15E-03
COL28A1	-3.15	0.05
KIFC2	-3.15	5.59E-06
FAM20A	-3.15	1.12E-03
SCNN1D	-3.14	4.94E-03
THRB	-3.14	1.66E-04
RGS17	-3.14	4.20E-04
P4HA1	-3.14	6.26E-07
HS3ST3B1	-3.14	0.01
IZUMO4	-3.13	0.03
AC135050.2	-3.12	9.02E-05
YJEFN3	-3.12	0.01
ACR	-3.12	1.83E-03
SMAD6	-3.11	2.93E-09
RIMKLA	-3.1	8.72E-07
WIPF3	-3.1	1.33E-09
MAP3K10	-3.1	9.37E-08
RHOU	-3.09	4.23E-06
KCNH8	-3.09	0.04
PFKFB4	-3.09	2.93E-10
HIVEP2	-3.09	1.14E-13
RNF112	-3.09	0.04
NHSL2	-3.09	0.04
NOTCH2NLB	-3.08	0.03
AL691442.2	-3.08	1.87E-10
NKX1-2	-3.08	0.03
SESN3	-3.08	8.78E-06
PRH2	-3.08	2.86E-03
NTN4	-3.08	2.15E-04
ONECUT1	-3.08	0.02

CLEC18B	-3.08	4.60E-03
PHLDB3	-3.08	6.96E-09
DCST2	-3.07	7.07E-04
NFASC	-3.07	5.03E-04
LRRC56	-3.07	9.92E-03
SRRM3	-3.06	1.67E-04
FAM166B	-3.06	0.04
SHC3	-3.06	0.01
MT-CO3	-3.06	3.03E-05
STRC	-3.05	2.82E-07
CD79B	-3.05	8.35E-03
CCDC88B	-3.04	2.73E-04
SCNN1B	-3.04	2.66E-03
CD164L2	-3.02	3.45E-04
CPA4	-3.01	1.18E-03
ACRBP	-3.01	8.23E-03
ARHGAP33	-3.01	1.41E-04
TCAF2	-3	2.00E-07
ZMIZ1	-3	8.68E-13
CHRD	-2.99	1.35E-06
HOXA13	-2.99	2.27E-07
AC022400.7	-2.99	0.03
HYPK	-2.99	3.27E-07
TMEM86B	-2.99	4.60E-03
AMT	-2.98	3.15E-06
MUC20	-2.98	9.05E-07
SEMA4B	-2.98	6.79E-17
MT-ND5	-2.98	2.39E-05
LURAP1L	-2.97	7.35E-03
TRIM69	-2.96	0.03
SLC17A7	-2.96	6.65E-03
AL512785.2	-2.95	1.25E-03
FAM110C	-2.95	3.76E-05
HES1	-2.95	4.34E-07
RGS14	-2.95	1.62E-06
PRICKLE1	-2.95	1.24E-04
COL18A1	-2.94	6.88E-14
RTN4RL1	-2.93	3.79E-03
FAM160A1	-2.92	1.57E-11
PIM1	-2.92	2.15E-09
MYO15B	-2.92	7.02E-08

GUCA1B	-2.91	3.29E-03
HOXA2	-2.91	8.56E-03
IFNLR1	-2.9	2.60E-07
TRIM46	-2.9	2.99E-03
TRIM9	-2.9	1.32E-07
FOXO6	-2.89	8.31E-05
ARID3B	-2.89	7.38E-07
SLC25A35	-2.89	9.81E-03
AMH	-2.89	2.63E-04
EPOR	-2.89	9.96E-10
ABCG1	-2.89	7.68E-04
MYOM1	-2.88	1.12E-04
GABRE	-2.88	7.80E-03
CPEB2	-2.87	1.10E-03
CDC42EP4	-2.87	1.32E-08
RNF165	-2.87	5.65E-07
MN1	-2.87	6.60E-06
TLR5	-2.86	2.23E-04
HERC3	-2.86	7.22E-10
FBN1	-2.86	3.99E-07
TFAP2C	-2.86	1.13E-06
BST1	-2.85	0.05
SMIM29	-2.85	1.06E-05
EDA	-2.85	9.08E-10
HECA	-2.84	3.29E-07
CASKIN1	-2.84	2.84E-05
HSH2D	-2.84	3.73E-07
RGS4	-2.83	0.04
MAPK15	-2.83	3.14E-07
CYP2C9	-2.83	0.02
ZBTB4	-2.83	2.44E-07
DIRAS3	-2.82	0.01
PPM1J	-2.82	8.02E-06
ABHD1	-2.82	0.04
NR4A1	-2.82	1.72E-09
AOC3	-2.82	1.28E-05
RIMS3	-2.81	2.96E-05
CCNG2	-2.81	1.34E-12
HOXC4	-2.81	7.56E-03
PRX	-2.81	2.91E-08
MT-ND2	-2.81	4.64E-04

IGFBP3	-2.8	0.03
MLLT3	-2.8	1.51E-07
ZMAT1	-2.8	1.20E-03
PDE5A	-2.79	1.12E-06
ANKRD37	-2.79	1.13E-06
AC026740.3	-2.79	5.23E-04
AFAP1L1	-2.79	2.78E-08
PPT2	-2.79	6.92E-06
SEMA3D	-2.79	9.07E-04
GOLGA8K	-2.79	1.96E-11
IRX3	-2.79	0.03
DENND1C	-2.79	1.01E-05
BCL11A	-2.78	5.06E-07
TTC9	-2.78	8.50E-05
GOLGA8H	-2.78	8.43E-09
ZNF469	-2.78	1.74E-08
MMP28	-2.78	0.01
TSSK3	-2.77	2.69E-03
CFAP126	-2.77	3.07E-03
PNMA2	-2.77	0.02
SPAG8	-2.77	0.05
FRMD4A	-2.77	2.47E-03
AL133352.1	-2.77	6.99E-05
LRRC4B	-2.77	1.69E-03
AC000093.1	-2.77	7.98E-06
FAM131C	-2.76	1.75E-07
THEMIS2	-2.76	1.84E-05
SYNGR3	-2.76	2.40E-03
ARL4D	-2.76	8.45E-06
EFNA4	-2.75	4.30E-03
C2orf72	-2.75	4.89E-03
TWIST1	-2.75	0.01
MARCHF9	-2.75	2.56E-05
PDLIM3	-2.74	1.44E-04
WHRN	-2.74	1.41E-07
SEC31B	-2.74	1.78E-06
NPAS1	-2.74	3.69E-03
TINAGL1	-2.73	6.06E-10
KIAA0040	-2.73	1.09E-10
GATA2	-2.73	1.37E-04
PHKG1	-2.73	0.03

PALM3	-2.73	4.69E-09
FOXD1	-2.72	1.18E-09
PKD2L1	-2.72	0.04
SH3PXD2A	-2.72	1.39E-07
CLK3	-2.72	2.40E-10
LPIN3	-2.72	4.53E-05
CCDC85A	-2.71	0.02
SLC25A45	-2.71	0.02
USP35	-2.71	2.62E-05
MBD6	-2.71	3.39E-05
SLC22A17	-2.71	2.94E-05
SLC26A11	-2.71	2.35E-07
ADAMTS10	-2.71	1.33E-04
ADGRB2	-2.7	6.12E-08
SLC15A2	-2.7	0.01
ELMOD1	-2.7	2.06E-03
ASPHD1	-2.7	8.09E-04
YBX2	-2.7	2.38E-03
NFATC2	-2.7	1.47E-08
SMIM1	-2.69	0.03
SIX3	-2.69	8.75E-05
ALPP	-2.69	0.03
TLX3	-2.69	1.81E-03
PRDM1	-2.69	0.03
ZNF395	-2.69	9.03E-07
NDRG1	-2.69	8.12E-09
DNAI1	-2.69	9.25E-04
CEACAM19	-2.69	0.03
C21orf58	-2.69	2.64E-03
NR3C2	-2.68	3.88E-05
TRIM39-RPP21	-2.68	0.03
AGFG2	-2.68	8.64E-09
AC068580.4	-2.68	7.09E-03
TRHDE	-2.68	0.05
BTBD11	-2.68	2.04E-05
SLC22A31	-2.68	5.58E-04
PAQR6	-2.67	8.18E-05
PLEKHA2	-2.67	5.87E-12
POLD4	-2.67	1.91E-04
PPP1R1A	-2.67	0.02
AC013394.1	-2.67	1.46E-03

CYTH4	-2.67	2.66E-05
PRDM16	-2.66	2.82E-07
TGFBR3	-2.66	1.08E-06
UBA7	-2.66	5.33E-03
ASIC3	-2.66	2.09E-04
SLCO4A1	-2.66	5.05E-12
TAS1R3	-2.65	0.04
SH2D5	-2.65	5.21E-03
GPC2	-2.65	1.45E-03
TMEM151A	-2.65	0.02
WNT7B	-2.65	6.05E-03
SLC25A27	-2.64	6.85E-05
LHX6	-2.64	3.95E-03
NFKB2	-2.64	3.65E-13
KREMEN2	-2.64	1.18E-04
RAPGEFL1	-2.64	8.48E-05
KRT13	-2.64	6.36E-03
PMEPA1	-2.64	0.01
LOXL2	-2.63	1.04E-09
PLEKHG6	-2.63	6.33E-08
IRS2	-2.63	3.70E-09
AHNAK2	-2.62	4.58E-06
EME2	-2.62	2.79E-03
MAST1	-2.62	1.54E-10
CYP4F12	-2.62	2.10E-03
APOL6	-2.62	0.05
LIFR	-2.61	2.39E-06
ZNF488	-2.61	2.83E-03
HSPB8	-2.61	4.45E-03
CCDC68	-2.61	2.28E-04
CCDC61	-2.61	5.66E-03
TSC22D4	-2.6	3.58E-09
QPCTL	-2.6	1.91E-07
MGLL	-2.59	6.24E-04
LCA5	-2.59	2.39E-04
C9orf131	-2.59	0.02
MXI1	-2.59	1.82E-10
PGGHG	-2.59	9.32E-05
NLRP14	-2.59	5.11E-03
PCSK4	-2.59	5.15E-03
BHLHE40	-2.58	9.02E-10

RNF182	-2.58	6.01E-04
GABBR1	-2.58	3.58E-07
RUNX2	-2.58	0.01
ADAMTS14	-2.58	1.24E-04
CHRM4	-2.58	7.32E-03
FNDC11	-2.58	0.01
NCMAP	-2.57	0.02
BACH2	-2.57	4.49E-05
HOXA1	-2.57	3.71E-03
ESRRB	-2.57	3.76E-04
GRAMD1A	-2.57	1.03E-12
PDE2A	-2.56	3.18E-05
RGCC	-2.56	0.03
LTK	-2.56	3.14E-04
THSD4	-2.56	2.63E-06
SCN4A	-2.56	3.27E-03
SLC5A5	-2.56	1.13E-03
PRPH	-2.55	3.25E-05
GALNT9	-2.55	0.04
NPIPB8	-2.55	5.33E-03
CARMIL2	-2.55	1.50E-07
CACNG8	-2.55	0.04
MEGF6	-2.54	6.59E-05
NRG2	-2.54	0.03
TEX19	-2.54	0.03
PCBP3	-2.54	0.03
AC068946.1	-2.53	8.15E-03
ETS1	-2.53	6.20E-04
C2CD4C	-2.53	0.03
APC2	-2.53	0.01
IQCN	-2.53	0.01
EML2	-2.53	1.07E-07
TNK2	-2.52	5.57E-07
PDZD2	-2.52	3.26E-03
USP2	-2.52	2.31E-05
CACNA1G	-2.52	7.20E-08
DACT3	-2.52	5.23E-03
OGFR	-2.52	2.21E-06
VILL	-2.51	0.01
CPM	-2.51	0.02
BICRA	-2.51	1.44E-04

HOXD8	-2.5	3.42E-04
PRRT4	-2.5	0.01
DENND3	-2.5	5.87E-10
CDH24	-2.5	1.01E-04
SMAD7	-2.5	4.58E-03
KLF2	-2.5	3.73E-07
LRRC75B	-2.5	1.34E-03
SAT1	-2.5	1.84E-04
SLC4A5	-2.49	1.88E-04
SATB1	-2.49	1.19E-09
FAT4	-2.49	3.34E-05
ZDHHC11B	-2.49	2.94E-03
MAST4	-2.49	1.58E-06
TNXB	-2.49	1.30E-10
GAB2	-2.49	2.72E-05
MRC2	-2.49	9.04E-07
SHANK1	-2.49	1.62E-06
MXRA8	-2.48	0.05
ARL4C	-2.48	6.02E-05
DDIT4L	-2.48	3.96E-03
TSPAN12	-2.48	9.58E-05
ADAMTS13	-2.48	2.68E-05
SCART1	-2.48	0.02
CCDC106	-2.48	0.04
CDK5R2	-2.47	0.02
B3GNT7	-2.47	4.58E-04
RAB43	-2.47	6.60E-04
MXD3	-2.47	2.57E-04
TRIM73	-2.47	4.40E-04
RASA4	-2.47	3.98E-07
TMEM86A	-2.47	7.71E-03
LRP1	-2.47	1.54E-05
TTBK2	-2.47	1.90E-09
PLA2G10	-2.47	4.88E-03
DLG4	-2.47	2.58E-07
NAA80	-2.46	7.50E-03
TENT5A	-2.46	1.38E-05
HOXB3	-2.46	1.32E-03
BAHCC1	-2.46	3.50E-06
TSHZ1	-2.46	1.93E-05
UPK1A	-2.46	0.01

MT-CO2	-2.46	3.31E-05
PIK3CD	-2.45	9.68E-11
GPR155	-2.45	8.06E-04
CYP21A2	-2.45	0.02
VSIG10L	-2.45	5.90E-03
LEMD1	-2.44	8.29E-05
TNFRSF10C	-2.44	1.13E-04
NEIL1	-2.44	1.85E-03
NAGS	-2.44	3.53E-06
FOSB	-2.44	3.11E-03
MED12L	-2.43	1.83E-06
PRDM8	-2.43	0.02
CEL	-2.43	9.35E-04
AP002990.1	-2.43	1.42E-03
BATF2	-2.43	0.02
S1PR5	-2.43	0.04
MRPL34	-2.43	5.96E-05
MYADM	-2.43	6.47E-08
ERFE	-2.42	1.32E-03
FKBP14	-2.42	1.82E-03
SLC26A4	-2.42	6.86E-03
CFAP157	-2.42	0.03
LMNTD2	-2.42	0.01
PPP1R32	-2.42	6.16E-05
NOD2	-2.42	2.09E-05
TCF7L1	-2.41	1.35E-06
PHF1	-2.41	1.90E-09
DNAJC25-GNG10	-2.41	0.03
C9orf16	-2.41	7.29E-03
PRSS8	-2.41	5.89E-09
SLC27A1	-2.41	1.23E-09
CHRNG	-2.4	0.04
SPRY1	-2.4	9.41E-04
KLHL32	-2.4	3.69E-03
IFITM1	-2.4	0.01
TSPAN18	-2.4	2.37E-05
SIRT4	-2.4	4.04E-03
SLC30A4	-2.4	4.02E-03
TBKBP1	-2.4	3.10E-05
KMT5C	-2.4	6.00E-05
NPR3	-2.39	0.05

FCHSD1	-2.39	1.57E-05
MTSS1	-2.39	1.61E-07
EGLN3	-2.39	2.90E-04
CCDC9B	-2.39	2.55E-03
FLYWCH1	-2.39	4.94E-05
SBSN	-2.39	7.18E-03
ACP7	-2.39	0.02
CYP24A1	-2.39	7.53E-04
DRP2	-2.39	0.04
MTMR11	-2.38	2.10E-04
SYNPO	-2.38	5.75E-09
PPP1R26	-2.38	3.38E-08
SH3BP1	-2.38	2.38E-09
SYNGR1	-2.38	1.46E-05
TMEM200B	-2.37	0.02
CYP26B1	-2.37	2.52E-05
SSC4D	-2.37	0.04
ADGRA2	-2.37	2.25E-06
GPR137	-2.37	0.01
PYGM	-2.37	5.92E-03
AP000812.5	-2.37	5.33E-03
PAPLN	-2.37	7.40E-03
HS3ST3A1	-2.37	3.29E-03
NFATC1	-2.37	4.71E-08
PRR36	-2.37	1.46E-05
COL4A4	-2.36	1.19E-06
HES6	-2.36	5.43E-04
KLHL24	-2.36	6.58E-08
AL365205.1	-2.36	2.22E-03
JAZF1	-2.36	2.75E-04
LIPH	-2.35	3.17E-08
IL18BP	-2.35	6.71E-03
ZNF385A	-2.35	3.25E-07
COL9A3	-2.35	5.65E-06
GAD1	-2.34	4.31E-05
GNRH1	-2.34	0.02
ADGRB1	-2.34	2.97E-04
SLC2A3	-2.34	2.91E-08
GCOM1	-2.34	3.60E-07
ANGPTL4	-2.34	4.11E-08
SEMA6C	-2.33	3.70E-05

MERTK	-2.33	6.80E-08
TMEM158	-2.33	0.01
PAG1	-2.33	1.26E-03
EXD3	-2.33	7.29E-04
AC012651.1	-2.33	0.02
FGF11	-2.33	0.01
MAFF	-2.33	0.01
KIF21B	-2.32	1.64E-08
ARID5B	-2.32	3.65E-09
RHEBL1	-2.32	4.12E-03
HAP1	-2.32	6.40E-03
TMEM91	-2.32	0.03
MAN1C1	-2.31	1.80E-03
PAQR8	-2.31	3.08E-04
CORO2A	-2.31	3.56E-06
ANKRD22	-2.31	2.52E-03
ULK1	-2.31	9.36E-07
GOLGA8J	-2.31	2.10E-04
PROSER3	-2.31	1.32E-04
NAT14	-2.31	1.04E-03
IQSEC2	-2.31	4.43E-08
RRAGD	-2.3	2.03E-05
AGPAT4	-2.3	2.91E-06
GPR37	-2.3	8.66E-03
MGAM2	-2.3	0.05
IL11RA	-2.3	7.25E-05
FAM78A	-2.3	0.01
ARHGEF17	-2.3	8.55E-08
IGFBP6	-2.3	6.98E-05
ITGA5	-2.3	2.15E-10
ZC2HC1C	-2.3	0.05
WNK4	-2.3	2.17E-05
RGS9	-2.3	3.01E-06
KLHL17	-2.29	1.54E-05
IER5	-2.29	6.62E-10
ANKRD23	-2.29	0.04
MGAT4A	-2.29	1.72E-03
NLRP1	-2.29	1.00E-04
DISC1	-2.28	0.05
TRIB2	-2.28	2.88E-06
KLHL29	-2.28	7.15E-06

TLL1	-2.28	0.02
TNIP1	-2.28	1.48E-08
MYEOV	-2.28	2.20E-06
SMAD3	-2.28	7.43E-11
LRRC3	-2.28	0.02
AKR7A3	-2.27	0.03
DMBX1	-2.27	0.03
LYST	-2.27	2.95E-07
KDM3A	-2.27	1.26E-09
KIF12	-2.27	2.86E-03
AKNA	-2.27	5.65E-07
MSS51	-2.27	0.03
PGM2L1	-2.27	7.41E-06
CEP126	-2.27	2.40E-04
CELF5	-2.27	1.62E-03
KDM4B	-2.27	2.54E-08
RAB40A	-2.27	0.01
DDIT4	-2.26	2.77E-06
NUTM2A	-2.26	1.17E-03
PDZD7	-2.26	8.16E-05
POLL	-2.26	3.48E-05
DUSP16	-2.26	6.42E-06
EPN2	-2.26	8.01E-08
PLEKHH2	-2.25	3.13E-07
SKIL	-2.25	5.53E-05
ACBD4	-2.25	3.26E-05
MYH7B	-2.25	0.03
EMID1	-2.25	1.10E-03
SYTL5	-2.25	0.02
CGN	-2.24	8.52E-10
HLA-F	-2.24	1.33E-08
GRIN2D	-2.24	4.52E-08
ID1	-2.24	2.00E-09
AGRN	-2.23	5.98E-05
GLIS3	-2.23	2.82E-03
ATG2A	-2.23	1.07E-07
JMJD7-PLA2G4B	-2.23	5.89E-03
KSR1	-2.23	9.87E-07
TBC1D3L	-2.23	1.48E-07
A4GALT	-2.23	4.22E-05
FRMD4B	-2.22	7.04E-04

CDKN2B	-2.22	0.02
SLC37A2	-2.22	0.02
POU6F1	-2.22	0.02
LPCAT4	-2.22	9.27E-09
TBC1D3I	-2.22	0.01
SPAG4	-2.22	0.01
AHDC1	-2.21	4.02E-07
METTL27	-2.21	1.65E-04
TMEM65	-2.21	5.33E-05
PELI2	-2.21	2.11E-04
MMP15	-2.21	5.76E-09
NOL3	-2.21	5.55E-03
TUBB8B	-2.21	0.04
SCN1B	-2.21	7.27E-04
Z83844.1	-2.21	4.19E-07
WNT9A	-2.2	0.03
MSX1	-2.2	1.90E-05
MLXIPL	-2.2	7.91E-05
MTRNR2L8	-2.2	0.03
SH2B3	-2.2	1.51E-06
TLE6	-2.2	4.90E-04
MARK4	-2.2	2.05E-09
CCDC30	-2.19	0.03
SOX13	-2.19	6.91E-08
ARHGEF28	-2.19	3.51E-05
PPP1R3B	-2.19	2.82E-05
SUSD3	-2.19	9.12E-03
KLF4	-2.19	1.85E-05
IGSF9B	-2.19	6.55E-05
VASH1	-2.19	3.61E-04
STARD9	-2.19	3.16E-06
FA2H	-2.19	7.62E-06
GATA6	-2.19	4.66E-08
EBF4	-2.19	2.90E-04
KIAA1522	-2.18	1.94E-05
DENND2C	-2.18	1.07E-03
TBX19	-2.18	6.51E-04
EPAS1	-2.18	3.54E-10
NKX3-1	-2.18	0.04
TRIQK	-2.18	2.32E-04
SKOR1	-2.18	0.03

GAS2L1	-2.18	2.91E-04
CCDC120	-2.18	2.20E-03
LIMS2	-2.17	0.04
GRID2IP	-2.17	0.02
GPR162	-2.17	0.02
ADCY6	-2.17	6.97E-07
TBC1D3B	-2.17	4.86E-05
TXNDC2	-2.17	0.03
AC011455.2	-2.17	1.86E-04
FAM110A	-2.17	0.02
PLEKHO1	-2.16	5.40E-04
KLF7	-2.16	7.58E-05
CREBRF	-2.16	3.92E-05
DNHD1	-2.16	2.48E-05
FOXO1	-2.16	5.98E-04
ENO3	-2.16	0.01
SLC16A13	-2.16	0.02
TMEM59L	-2.16	1.27E-05
FZD7	-2.15	2.63E-05
SNX25	-2.15	2.18E-07
N4BP3	-2.15	0.01
FAM167A	-2.15	0.02
DNAJB5	-2.15	4.91E-05
CDHR5	-2.15	0.01
TCP11L2	-2.15	1.45E-05
CLDND2	-2.15	0.04
SLC41A1	-2.14	6.19E-09
COL4A3	-2.14	1.05E-06
ITIH4	-2.14	0.03
ILDR1	-2.14	8.90E-04
SGK1	-2.14	9.46E-03
KCNH2	-2.14	3.52E-07
EPPK1	-2.14	3.50E-06
PTGS1	-2.14	4.59E-03
VSIR	-2.14	9.49E-03
CALB2	-2.14	0.01
ABCA7	-2.14	3.33E-06
TJP3	-2.14	4.68E-09
SULT2B1	-2.14	3.29E-03
HS1BP3	-2.13	9.72E-06
ZNF516	-2.13	7.16E-07

PPARG	-2.12	1.70E-03
CHSY3	-2.12	0.01
TRIM15	-2.12	0.01
TANC2	-2.12	1.87E-08
FSTL3	-2.12	1.39E-03
TP53INP2	-2.12	0.04
RTEL1-TNFRSF6B	-2.12	1.71E-05
CBX7	-2.12	0.03
IL1RAPL1	-2.12	5.09E-03
SEMA3F	-2.11	1.30E-06
RAB6B	-2.11	8.22E-05
SHOX2	-2.11	2.98E-03
ABLIM3	-2.11	3.94E-08
FBXO32	-2.11	7.54E-04
FRMPD1	-2.11	0.01
OAF	-2.11	1.08E-03
CTDSP2	-2.11	9.45E-09
OCEL1	-2.11	1.12E-04
SRRM5	-2.11	2.48E-03
CYP2D6	-2.11	0.02
RNF207	-2.1	2.54E-03
FBXO44	-2.1	2.90E-04
ATP6V1B1	-2.1	0.04
C3orf18	-2.1	1.66E-03
PAX6	-2.1	1.76E-04
ELK3	-2.1	5.59E-08
MSLN	-2.1	2.83E-06
CNTNAP1	-2.1	3.68E-03
EVI5L	-2.1	7.30E-08
LZTS3	-2.1	0.02
MAP3K15	-2.1	1.12E-03
DDR2	-2.09	0.03
BVES	-2.09	0.02
FBXO24	-2.09	0.03
CCDC136	-2.09	1.24E-03
TESMIN	-2.09	0.01
CACNB3	-2.09	1.47E-07
STARD5	-2.09	0.02
ZMYND15	-2.09	0.03
PLIN4	-2.09	6.66E-03
CPT1B	-2.09	9.27E-04

FRMPD3	-2.09	3.70E-03
KIF3C	-2.08	6.35E-05
TMEM42	-2.08	2.08E-03
FAM131A	-2.08	2.51E-03
MUC12	-2.08	0.02
ZNF775	-2.08	6.17E-03
LGR4	-2.08	4.44E-04
PPP2R5B	-2.08	1.86E-06
PHETA1	-2.08	0.02
RHOV	-2.08	0.02
PML	-2.08	3.88E-06
HAPLN3	-2.08	1.61E-04
SH2B1	-2.08	5.31E-04
CTIF	-2.08	9.25E-06
MAP4K1	-2.08	3.01E-03
CDKN1A	-2.07	2.31E-06
NEURL1	-2.07	0.03
REC8	-2.07	0.03
ST3GAL2	-2.07	6.58E-03
PPP1R1B	-2.07	0.03
PLEKHH3	-2.07	8.24E-04
CCBE1	-2.07	0.03
TOX2	-2.07	7.61E-07
KCND1	-2.07	3.90E-03
FOXO4	-2.07	6.30E-05
GALNT3	-2.06	4.27E-04
RELL2	-2.06	4.80E-03
ZNF783	-2.06	3.76E-03
FIBCD1	-2.06	1.42E-03
CACNB2	-2.06	3.14E-03
ZNF774	-2.06	0.03
RRAS	-2.06	4.03E-04
ZSCAN20	-2.05	7.14E-04
ERO1B	-2.05	2.18E-03
MXD1	-2.05	3.80E-03
SLC26A6	-2.05	1.22E-04
C3orf67	-2.05	3.81E-04
LHFPL2	-2.05	2.03E-03
TNFAIP8	-2.05	1.68E-04
AL358113.1	-2.05	0.03
FBRS	-2.05	9.37E-05

KIAA0895L	-2.05	7.31E-03
AC092073.1	-2.05	0.04
DEDD2	-2.05	1.38E-03
RNF215	-2.05	3.96E-04
TENT5C	-2.04	0.03
PDK4	-2.04	4.46E-03
TRPV6	-2.04	2.49E-03
BX664615.2	-2.04	0.02
ENTPD2	-2.04	1.23E-04
PIDD1	-2.04	4.46E-03
ROBO3	-2.04	3.05E-03
SPSB2	-2.04	0.01
ITGA7	-2.04	1.24E-04
MMP17	-2.04	1.12E-03
GOLGA6L10	-2.04	5.26E-06
CAMTA2	-2.04	3.00E-07
TLE2	-2.04	2.69E-05
AC024592.3	-2.04	1.37E-03
CDK18	-2.03	4.00E-07
ARHGAP31	-2.03	2.03E-04
USP17L11	-2.03	3.24E-03
STOX2	-2.03	1.35E-03
FCHO2	-2.03	2.58E-07
CYSTM1	-2.03	2.17E-04
TMUB1	-2.03	5.86E-03
TMC1	-2.03	0.02
GATA3	-2.03	8.18E-04
CSPG4	-2.03	1.72E-04
C16orf74	-2.03	0.04
CORO6	-2.03	2.10E-03
CIC	-2.03	8.75E-05
ZBTB46	-2.03	0.02
SLC27A3	-2.02	1.42E-03
PILRB	-2.02	4.27E-03
ZNF862	-2.02	1.80E-04
CA9	-2.02	0.02
EHBP1L1	-2.02	2.32E-08
TMPRSS13	-2.02	9.70E-03
LDHD	-2.02	0.04
ADAM11	-2.02	6.24E-04
BRSK1	-2.02	5.56E-05

TRIOBP	-2.02	3.79E-08
TENT5B	-2.01	8.98E-03
ABTB1	-2.01	2.75E-04
RGL2	-2.01	1.30E-04
SH3BGRL2	-2.01	1.20E-03
PPP1R35	-2.01	7.21E-04
UNC5B	-2.01	7.26E-07
FAM234B	-2.01	7.60E-06
TUBG2	-2.01	1.78E-07
PTK6	-2.01	0.02
CD55	-2	5.57E-03
TTLL3	-2	2.91E-03
HLA-E	-2	9.37E-08
VGF	-2	2.26E-03
ZBTB10	-2	6.25E-07
ST3GAL1	-2	2.90E-03
OLFML2A	-2	1.28E-03
IRF7	-2	0.03
NOTCH3	-2	1.40E-07
LRFN3	-2	4.39E-03
ZNHIT2	2	0.05
POLR3B	2	9.86E-08
PKNOX1	2	7.71E-05
B3GALNT1	2.01	4.50E-04
GTF2IRD2B	2.01	2.22E-07
ZNF485	2.01	3.49E-04
SLC25A16	2.01	3.01E-04
THAP11	2.01	5.96E-05
EXOSC6	2.01	9.37E-04
UBTF	2.01	8.19E-08
RDH13	2.01	8.67E-06
ZNF587	2.01	4.22E-07
RBM12	2.01	1.73E-07
ANAPC13	2.02	3.00E-04
AL132671.2	2.02	4.64E-03
BCL7B	2.02	6.89E-07
TGS1	2.02	1.05E-07
UTP20	2.02	1.82E-08
ZNF649	2.02	4.01E-05
FN1	2.03	0.01
TAF9	2.03	4.49E-06

AC068775.1	2.03	1.22E-05
MRPS11	2.03	3.44E-07
CEBPA	2.03	1.47E-03
ZNF551	2.03	3.93E-06
MRPL20	2.04	4.10E-08
LIPT1	2.04	0.03
CDKN2AIP	2.04	1.81E-04
H4C2	2.04	5.15E-07
ANGPT2	2.04	0.02
WDR76	2.04	8.38E-08
AEN	2.04	3.25E-06
CES1	2.04	0.05
FAAP24	2.04	0.02
YWHAH	2.04	2.25E-08
SRSF10	2.05	1.31E-07
TAF13	2.05	5.34E-04
NMI	2.05	1.96E-03
RPP40	2.05	2.58E-04
ZBTB24	2.05	1.11E-07
KLHL42	2.05	1.00E-05
POLR3K	2.05	6.17E-04
TADA2A	2.05	2.22E-05
CDC6	2.05	2.62E-08
SFT2D2	2.06	3.49E-08
ODC1	2.06	6.46E-07
PPIL1	2.06	1.07E-07
TUBA8	2.06	5.91E-03
MAGOH	2.07	4.69E-06
EPM2AIP1	2.07	6.12E-04
BTC	2.07	0.03
UTP15	2.07	3.49E-07
KLRC4	2.07	0.04
PYROXD1	2.07	1.54E-04
LLPH	2.07	1.78E-04
GINS3	2.07	4.02E-05
DDX28	2.07	1.44E-05
SRPRB	2.08	7.00E-08
H2AC6	2.08	6.10E-07
H2AC12	2.08	8.69E-06
ASF1A	2.08	5.62E-08
CCNJ	2.08	4.56E-07

WDR73	2.08	6.01E-04
ZNF669	2.09	6.35E-04
PDF	2.09	0.02
TRIM16	2.09	5.52E-09
HOXB7	2.09	7.68E-06
DDX5	2.09	7.63E-08
ADNP	2.09	3.46E-07
ZNF124	2.1	1.02E-06
NIFK	2.1	3.38E-05
TRANK1	2.1	0.02
AHRR_1	2.1	1.72E-04
RAB19	2.1	1.89E-03
ZNF268	2.1	1.61E-04
ALG5	2.1	5.80E-07
AJUBA	2.1	7.96E-07
C3	2.1	0.02
HMGN2	2.11	5.20E-08
AL713999.1	2.11	1.89E-03
RRP36	2.11	5.00E-08
DNAJA1	2.11	5.44E-08
TOR1A	2.11	1.92E-08
PRDM15	2.11	1.27E-07
H3C15	2.12	2.93E-06
H3C2	2.12	1.11E-07
DDX31	2.12	5.13E-09
ZNF544	2.12	9.41E-07
WDR77	2.13	5.34E-06
H2BC18	2.13	5.18E-07
DTL	2.13	2.01E-07
H4C3	2.13	1.53E-08
ORC5	2.13	3.46E-07
TMEM138	2.13	1.11E-03
ZBTB1	2.13	2.03E-08
DERPC	2.13	7.04E-07
CCNE1	2.13	9.89E-09
SRSF6	2.13	1.12E-08
SCO2	2.13	2.87E-08
ABHD5	2.14	6.45E-07
SLC25A13	2.14	6.66E-07
AKR1C2	2.14	0.02
CTCF	2.14	3.35E-10

MIS12	2.14	1.13E-04
GPCPD1	2.14	7.11E-04
TRMT10C	2.15	1.97E-07
IDNK	2.15	0.04
RBM4	2.15	6.02E-09
POLE2	2.15	8.58E-07
AC010522.1	2.15	0.01
ADI1	2.16	9.17E-05
NOP56	2.16	1.08E-08
NXT1	2.16	3.54E-07
NUP50	2.16	4.89E-10
CCDC121	2.17	9.51E-03
AL031777.2	2.17	3.93E-06
ZBTB2	2.17	2.50E-06
MCM10	2.17	5.71E-08
OBI1	2.17	1.84E-08
SERPINA1	2.17	7.68E-03
ZNF587B	2.17	1.47E-06
BCLAF1	2.18	1.12E-09
ZNF239	2.18	6.42E-05
RCBTB2	2.18	0.02
ZNF708	2.18	8.79E-08
HAVCR1	2.19	0.05
PLD6	2.19	4.45E-03
PSMC3IP	2.19	7.74E-09
PDE12	2.2	6.15E-08
H2BC4	2.2	2.97E-06
ZNF787	2.2	1.46E-03
DCLRE1A	2.21	2.72E-08
DNAJC9	2.22	3.12E-09
AC008012.1	2.22	3.14E-04
CT45A5	2.22	0.04
COA7	2.23	2.76E-07
RNF103-CHMP3	2.23	1.48E-04
WDR5B	2.23	1.78E-03
ORM1	2.23	6.95E-04
SLC35G1	2.23	7.45E-04
NR2F2	2.23	1.75E-08
NIP7	2.23	1.05E-09
NOL12	2.23	8.87E-05
ANKRD16	2.24	1.46E-05

RBM4B	2.24	2.66E-09
TLCD1	2.24	1.30E-04
ZNF724	2.24	1.82E-09
PRR19	2.24	0.05
GRWD1	2.24	1.65E-09
PCNA	2.24	1.72E-09
MRTO4	2.25	5.39E-10
AC005154.5	2.25	3.71E-03
CHST3	2.25	2.27E-06
FDXACB1	2.25	5.30E-04
TEN1	2.25	3.65E-04
STS	2.25	1.18E-03
CDC25A	2.26	7.13E-08
TRMT6	2.26	2.43E-09
RABIF	2.27	6.53E-03
PNO1	2.27	2.86E-07
NFXL1	2.27	1.81E-08
H3C1	2.27	2.35E-06
METTL1	2.27	5.84E-07
ZNF26	2.27	7.81E-04
KEAP1	2.27	9.18E-08
KTI12	2.28	6.23E-03
CENPS	2.29	8.13E-03
ISY1-RAB43	2.29	5.82E-03
GTF3C6	2.3	5.72E-06
LTV1	2.3	5.81E-09
MAGOHB	2.3	2.20E-08
HNF4A	2.3	0.03
AC092647.5	2.31	3.93E-03
PAH	2.31	1.59E-03
RBL1	2.31	4.96E-08
TTC32	2.32	0.04
AC079447.1	2.32	0.04
RCHY1	2.32	3.76E-05
TLR3	2.32	0.03
ORM2	2.32	2.45E-03
A2M	2.32	0.02
AKR1C1	2.33	4.93E-03
CENPS-CORT	2.34	2.02E-05
ZBTB8A	2.34	0.02
POLE3	2.34	1.34E-10

TRIM16L	2.34	6.65E-09
H2BC11	2.35	1.82E-07
ITIH2	2.35	0.01
AC093227.2	2.35	0.02
TM4SF1	2.37	0.03
PTGES3L-AARSD1	2.37	4.68E-03
NCOA5	2.37	6.76E-05
NOTCH2NLA	2.38	0.01
SMIM15	2.38	2.53E-09
ZC3HAV1L	2.38	1.96E-04
TMEM250	2.38	8.50E-04
SLC43A3	2.38	3.02E-10
UBIAD1	2.4	3.55E-11
H2AC11	2.4	1.34E-09
CLSPN	2.41	1.59E-11
MAK16	2.41	7.49E-10
PMM2	2.41	1.99E-10
ARSL	2.41	0.04
ZNF367	2.42	5.45E-07
ABITRAM	2.42	7.01E-06
FKRP	2.42	1.36E-03
EPC2	2.43	4.04E-11
IFIT2	2.43	2.68E-03
EEF1AKMT1	2.43	6.29E-06
ZFX	2.43	9.53E-11
SRP14	2.44	1.81E-06
H2AC14	2.45	4.19E-07
SIK1B	2.45	0.04
TAF9B	2.45	2.90E-09
H3C14	2.47	3.69E-07
GPER1	2.47	8.84E-03
PPIF	2.47	3.27E-12
MKRN2OS	2.48	0.05
EPM2A	2.48	8.66E-05
HSPA8	2.48	2.29E-11
SLC16A4	2.49	0.05
CHAC2	2.49	2.75E-04
PDE4B	2.5	3.01E-08
H1-6	2.5	0.02
LCMT2	2.5	2.72E-06
TXNL4B	2.5	1.13E-04

TRPM2	2.5	2.32E-08
H2AC18	2.51	2.98E-08
AGT	2.51	7.92E-03
PCDHB7	2.51	2.92E-03
RFXAP	2.51	1.42E-06
PRMT6	2.52	3.87E-03
APOA2	2.52	6.03E-03
H4C8	2.52	1.75E-05
LYZ	2.52	4.65E-03
HRG	2.53	9.76E-03
BEND3	2.53	3.04E-05
ASTE1	2.54	1.97E-04
AC084337.2	2.54	3.46E-04
MT1E	2.54	5.90E-10
FAIM	2.55	9.32E-08
CNGB1	2.55	0.02
SLC7A11	2.57	4.82E-09
INMT-MINDY4	2.59	2.00E-03
GRIN2B	2.61	1.51E-07
PIK3R3	2.62	0.01
MAT2A	2.62	3.23E-14
SHLD3	2.62	0.02
H2AC4	2.62	3.63E-10
TIPIN	2.62	2.43E-10
GK3P	2.63	0.01
OAS1	2.63	0.04
TIMM8B	2.64	8.85E-05
NLRP11	2.64	2.66E-03
RPS4Y1	2.65	9.79E-03
H2BC14	2.66	8.48E-06
FAM111B	2.67	9.19E-06
SERPINB8	2.68	6.18E-07
H2AW	2.69	2.43E-11
SETMAR	2.71	1.01E-09
TLCD3A	2.72	5.29E-12
C1orf131	2.76	1.26E-10
LYRM2	2.77	4.76E-06
ZNF804A	2.78	0.02
TRMO	2.79	2.14E-08
EPC1	2.79	7.23E-14
SRSF7	2.8	1.19E-15

RRS1	2.8	1.30E-10
MCTS2P	2.8	1.36E-03
CCN1	2.83	9.41E-15
LANCL2	2.85	1.78E-09
H4C4	2.86	6.90E-14
ZNF114	2.86	4.90E-06
CCT6B	2.87	4.00E-03
PBDC1	2.87	4.44E-14
PCDHB12	2.89	0.05
RPL36A-HNRNPH2	2.9	8.02E-08
SFPQ	2.91	1.04E-15
ALB	2.91	9.96E-04
POU5F1B	2.92	1.39E-03
ODAM	2.94	0.03
TIGD6	2.94	1.96E-04
H2AC8	2.95	3.77E-03
URB2	2.97	1.72E-14
GPX2	2.99	0.02
PIGW	3	1.20E-09
GPATCH4	3.03	6.34E-15
H2AC19	3.05	1.09E-09
MFSD4A	3.07	0.05
STIMATE	3.07	0.03
PAK1IP1	3.07	1.05E-10
ALDH1A1	3.07	8.15E-03
PCDHA10	3.09	6.27E-04
SPP1	3.1	7.27E-04
H2AC15	3.1	3.55E-05
TIGAR	3.12	1.86E-03
H3C12	3.15	1.54E-07
H2BC8	3.17	3.58E-03
CNTD2	3.18	0.03
IFIT3	3.2	7.59E-03
CHST4	3.22	0.03
AC055811.2	3.23	8.90E-05
ZSCAN31	3.25	4.36E-09
SLC16A9	3.26	4.67E-06
HPD	3.27	4.77E-04
CDH2	3.3	0.02
CCNE2	3.32	6.48E-09
TAS2R30	3.33	4.55E-04

PHYHIPL	3.39	0.04
LETM2	3.41	2.19E-18
PCDHB16	3.42	0.04
ELMO1	3.47	1.99E-03
AC036214.3	3.48	0.01
WDR92	3.49	6.47E-04
AL357075.4	3.49	4.86E-03
SERPINA6	3.49	3.84E-03
H4C15	3.51	8.82E-03
SDC2	3.52	0.02
HYDIN	3.53	0.05
AKR1B10	3.55	3.07E-04
MKX	3.55	8.53E-15
WDR93	3.57	0.03
LAMA2	3.59	1.44E-11
AC104389.6	3.6	3.42E-03
PPAN-P2RY11	3.62	1.86E-05
H3C3	3.7	9.15E-06
PIEZO2	3.71	0.01
FEZ1	3.72	0.05
ACRV1	3.72	0.02
KDM5D	3.73	0.02
SLC16A6	3.78	6.48E-11
UGT2B7	3.82	1.79E-03
PBLD	3.84	2.77E-05
AGMAT	3.86	0.03
SOX5	3.86	0.05
SPART	3.86	0.03
MYO3B	3.91	0.03
TOE1	3.94	1.15E-14
KRT35	3.95	8.58E-03
DSG4	3.95	0.02
APOB	3.97	1.55E-05
HCN1	3.99	5.43E-06
DDC	3.99	0.02
AC068631.3	4.06	0.05
KRT36	4.09	0.04
SERPINA7	4.1	6.73E-03
MANSC4	4.2	2.17E-03
APOH	4.25	3.68E-04
ABCG5	4.39	0.03

H2AC16	4.4	0.01
ZC3H11B	4.42	4.64E-03
CHFR	4.49	0.04
SCCPDH	4.56	0.01
URGCP-MRPS24	4.56	5.32E-04
SPATA12	4.61	6.85E-03
HP	4.61	1.71E-04
VLDLR	4.65	0.01
AC008073.3	4.72	0.02
GJB1	4.72	0.01
LONRF2	4.74	7.17E-05
SLC7A7	4.75	5.48E-03
ITGA11	4.75	0.05
TBX15	4.81	0.02
SLC40A1	4.82	0.01
GSAP	4.88	8.91E-03
KRT37	5.03	0.04
CRPPA	5.06	1.75E-04
APOA4	5.08	0.03
CHRM1	5.23	8.07E-04
AC008764.4	5.35	9.51E-03
ASPN	5.39	0.01
ACOT1	5.41	1.76E-03
AC004805.1	5.45	8.24E-03
PCDHGB1	5.51	2.74E-03
CD96	5.68	0.03
CPLX2	5.73	1.17E-04
FAM187A	5.75	0.04
ZYG11A	5.79	0.05
ACY3	5.79	0.05
FBN2	5.92	0.02
AL645922.1	5.99	8.31E-03
GBP1	6.17	0.02
ZNF556	6.29	4.00E-03
CCNI2	6.33	5.26E-03
RIPOR3	6.35	0.04
AL109811.3	6.53	8.72E-03
AC004706.3	6.55	0.02
SPINK1	6.72	0.02
PCDHA4	6.88	0.04
ZNF626	6.9	0.05

NUGGC	6.91	0.02
HPR	6.93	2.55E-03
C7orf25	7.16	5.46E-03
FAM243A	7.18	0.02
TAS2R13	7.45	0.03
SV2A	7.48	0.02
CUZD1	8.04	6.35E-03
ADGRL2	8.05	2.06E-03
H2BC3	8.06	1.13E-05
SORBS2	8.1	0.03
SAA4	8.13	4.87E-03
OMD	8.27	0.02
UGT1A6	8.39	0.05
AC048338.1	8.52	2.82E-04
SULT2A1	8.85	2.10E-03
FAM47E	8.93	0.03
GNA15	8.93	0.04
NUPR1	9.26	0.02
PCDHB3	9.3	1.58E-03
KRTAP4-16	9.45	2.92E-03
MYLK3	9.48	0.04
HAAO	9.53	0.02
NRXN1	9.58	0.04
AL136295.5	9.87	5.54E-05
KIRREL3	9.88	0.03
SHPK	10.16	8.34E-18
ANKUB1	10.18	0.03
C6orf58	10.59	6.48E-03
BAAT	10.83	1.23E-03
RAB32	10.91	0.02
AC008403.1	11.15	5.37E-03
SLAMF7	11.35	1.64E-03
KRTAP4-9	11.4	3.65E-03
H1-1	11.96	3.74E-03
SLC22A3	12.28	8.30E-04
F7	12.79	0.02
ZFY	13.44	4.76E-03
CCL15	14.36	0.01
UGT2A3	15.48	1.03E-04
PCDHA8	17.54	4.22E-03
TM4SF19	19.1	5.52E-03

OR10A5	20.71	3.27E-03
PCDHB5	27.17	0.05
NXPE1	27.17	0.05
HSPA6	29.57	0.05
DMRTC1	30.12	0.05
SPATA18	30.18	0.04
CCKAR	30.42	0.04
RLBP1	30.43	0.04
GNGT2	30.43	0.04
GFRA2	30.83	0.04
MOGAT2	32.86	0.04
LYNX1-SLURP2	33.53	0.04
KRTAP5-6	33.53	0.04
FANCD2OS	33.68	0.03
CLNK	33.68	0.03
GRM7	33.69	0.03
BRD3OS	33.69	0.03
ADH4	34.67	0.04
AL355312.6	34.83	0.05
AC015813.2	34.83	0.05
ALPI	35.58	0.04
AKR1B15	35.77	0.04
AC087289.3	35.77	0.04
OTOGL	36.95	0.03
AC008481.3	37.8	0.03
CROT	37.95	0.03
MPDZ	38.34	0.04
TULP2	40.23	0.03
NLRP9	40.59	0.03
DNAH7	40.6	0.03
MRAP	42.62	0.03
TEX28	43.07	0.03
SLC5A9	44.63	0.03
SOX10	46.74	0.02
ACSM2B	48.12	0.02
AC048338.2	48.65	0.03
BIVM-ERCC5	54.95	0.02
CRYAA	59.48	0.02
PLA2G4B	65.4	0.02
AL451062.4	71.9	0.01
UBD	77.18	0.01

MAGED4	90	0.01
HSFX2	131.45	2.66E-03
AC008695.1	157.28	3.71E-03
ARL2-SNX15	175.41	3.01E-03
AC078927.1	233.03	8.88E-04
GP1BB	269.09	8.83E-04
UPK3BL1	299.19	1.16E-03

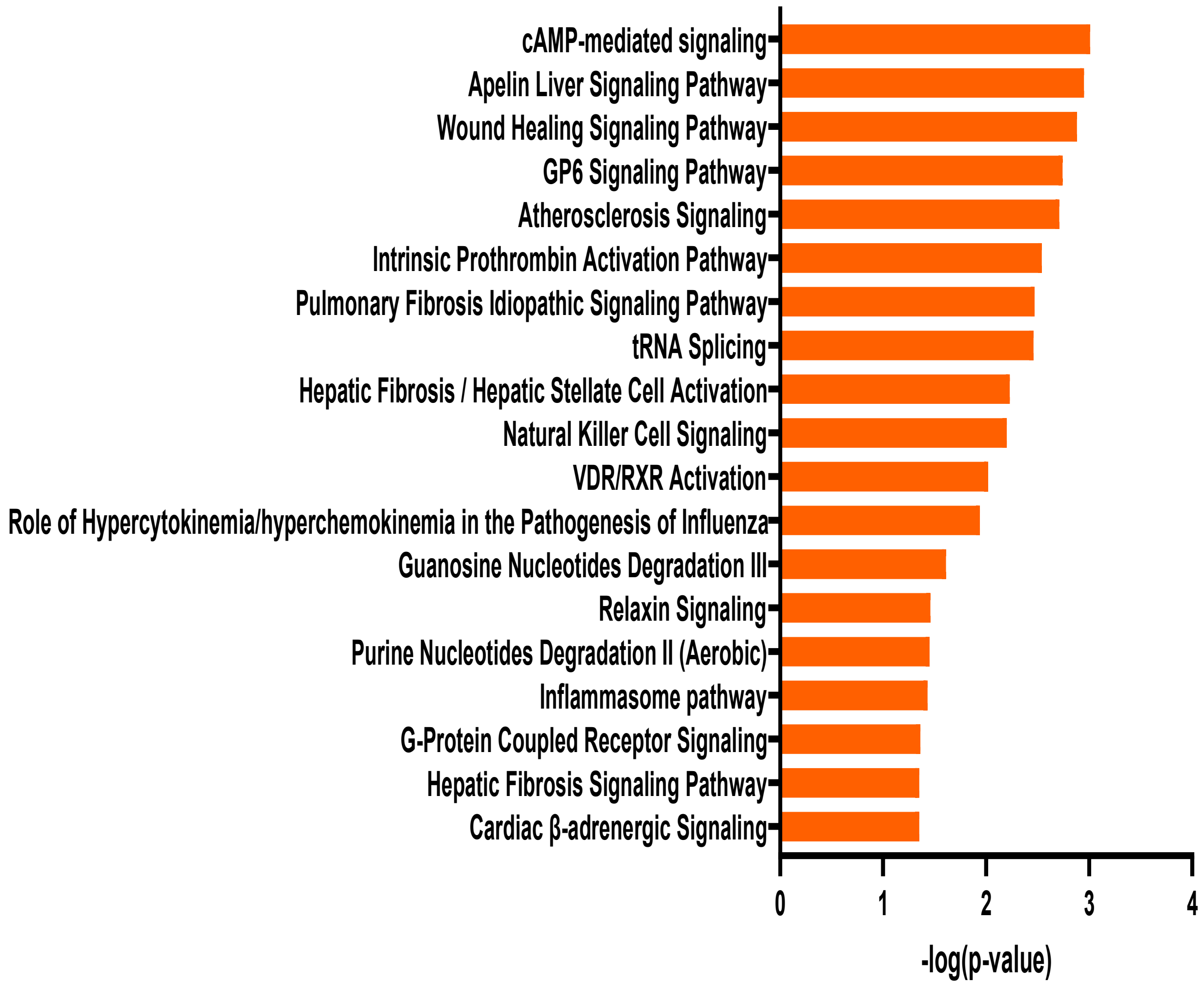

Figure S1. Significant canonical pathways identified by Ingenuity Pathway Analysis (IPA) when using the upregulated DEGs between steatotic and untreated cells

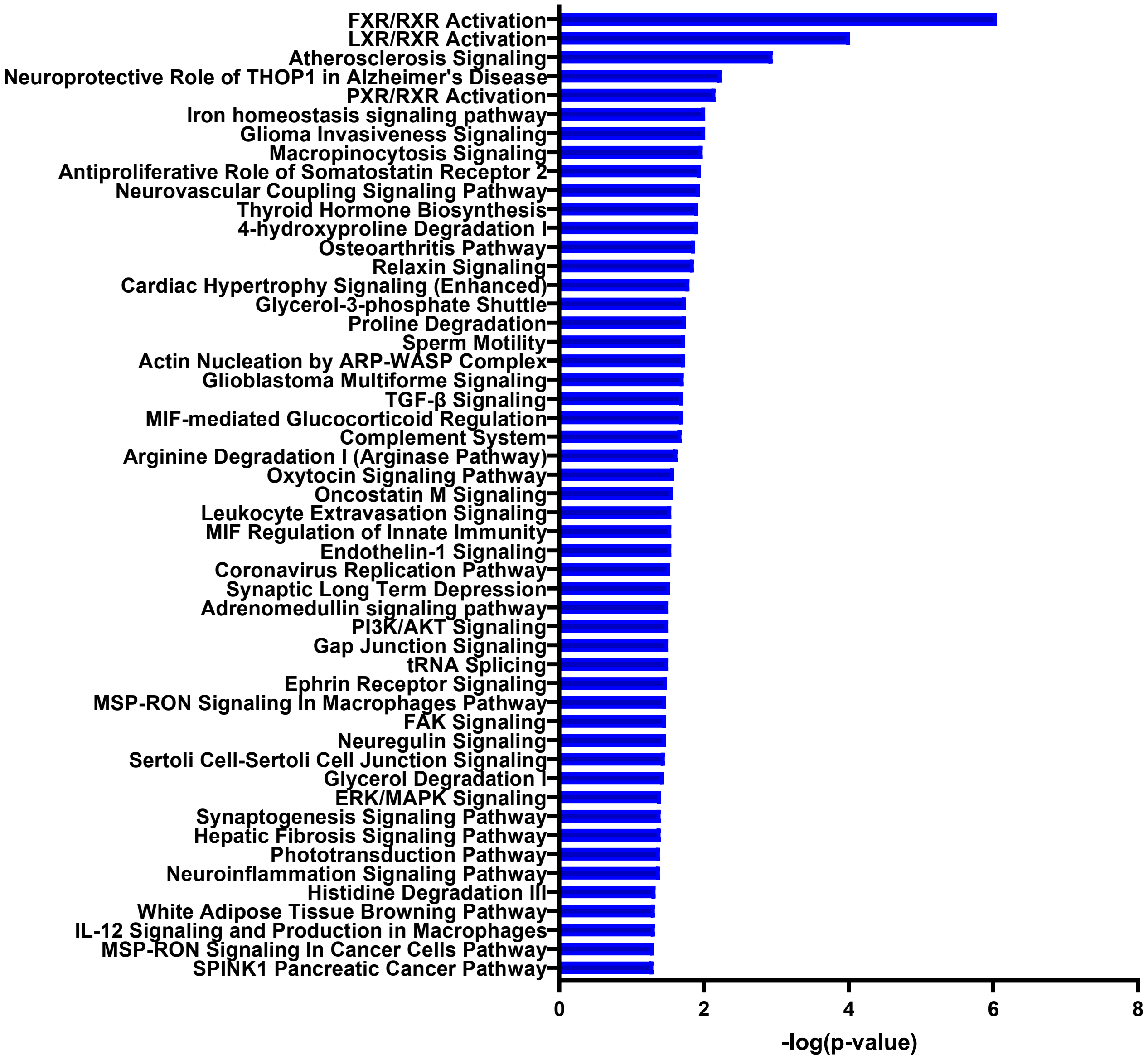

Figure S2. Significant canonical pathways identified by Ingenuity Pathway Analysis (IPA) when using the downregulated DEGs between steatotic and untreated cells.

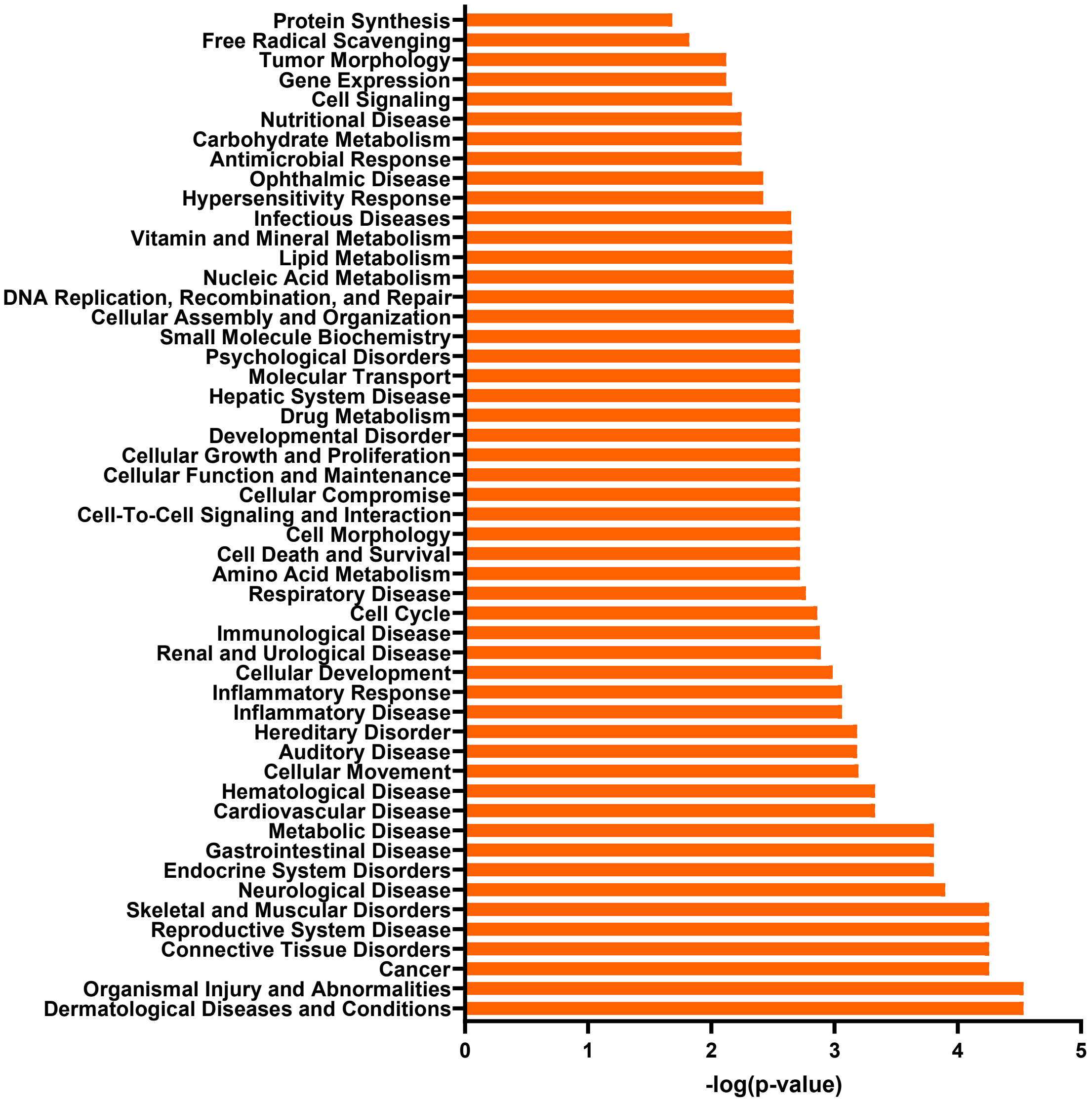

Figure S3. *Significant Molecular and Cellular Functions identified by Ingenuity Pathway Analysis (IPA) when using the upregulated DEGs between steatotic and Ucs.*

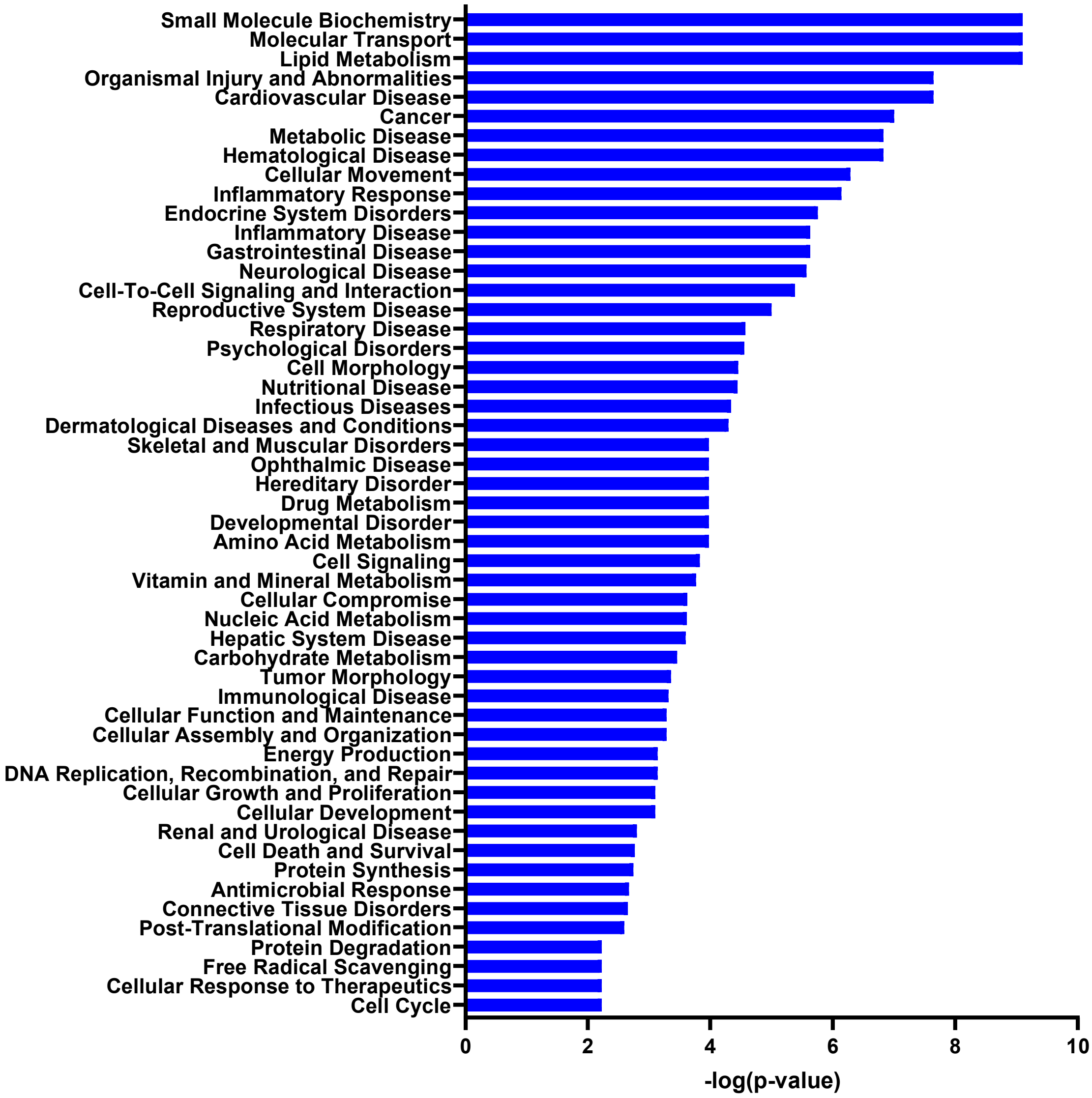

Figure S4. *significant Molecular and Cellular Functions identified by Ingenuity Pathway Analysis (IPA) when using the doenregulated DEGs between steatotic and UCs.*

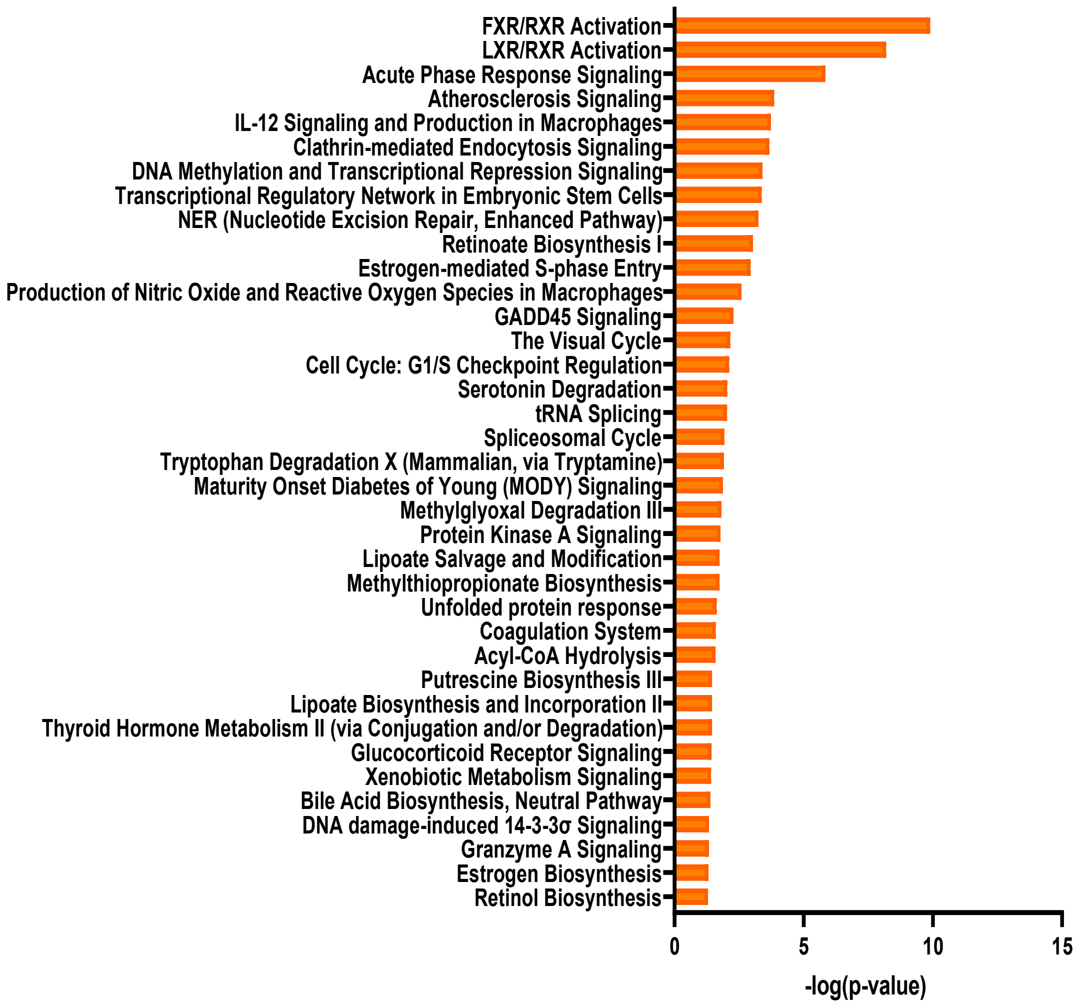

Figure S5. *Significant canonical pathways identified by Ingenuity Pathway Analysis (IPA) when using the upregulated DEGs between steatotic and Ex-4-treated steatotic cells*

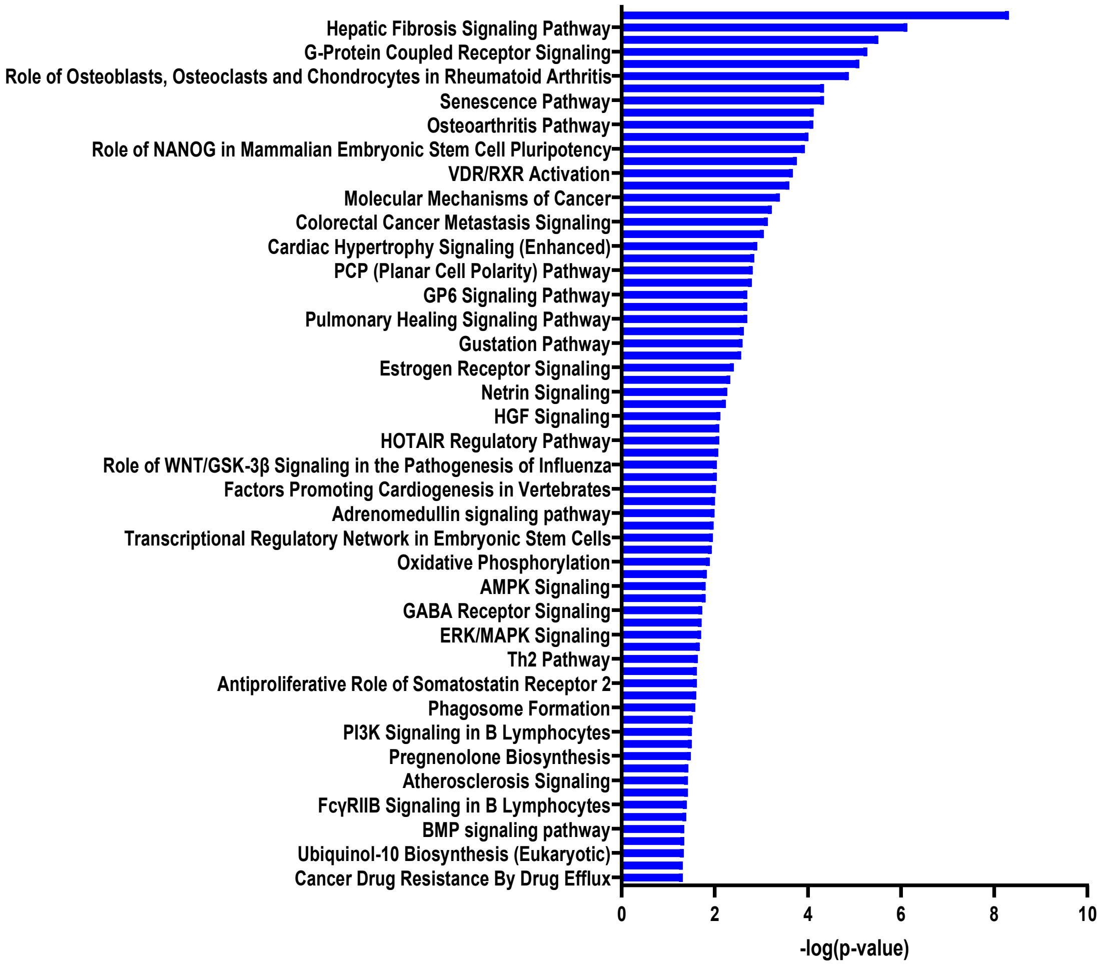

Figure S6. *Significant canonical pathways identified by Ingenuity Pathway Analysis (IPA) when using the downregulated DEGs between steatotic and Ex-4-treated steatotic cells*

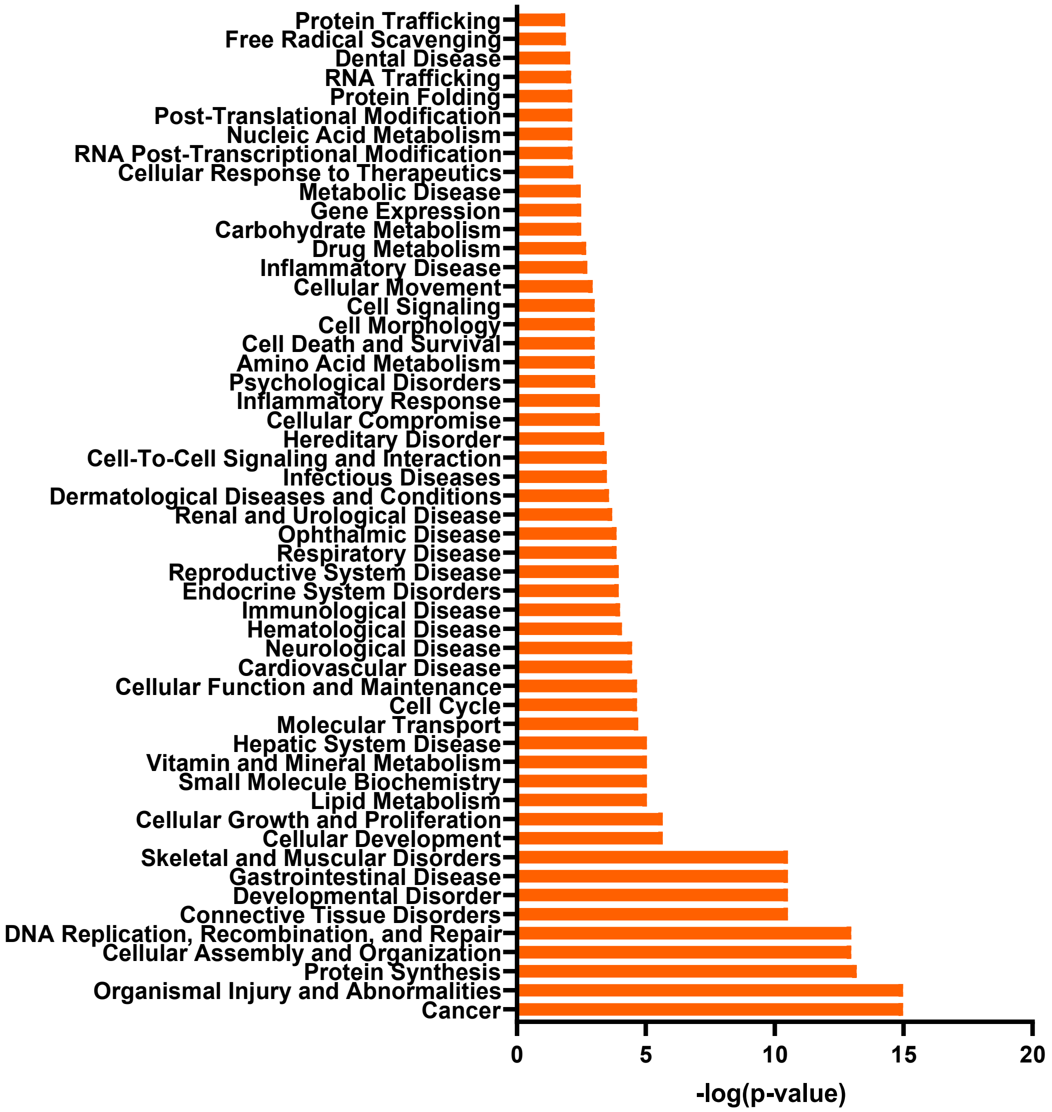

Figure S7. *Significant Molecular and Cellular Functions identified by Ingenuity Pathway Analysis (IPA) when using the upregulated DEGs between steatotic and Ex-4-treated steatotic cells*

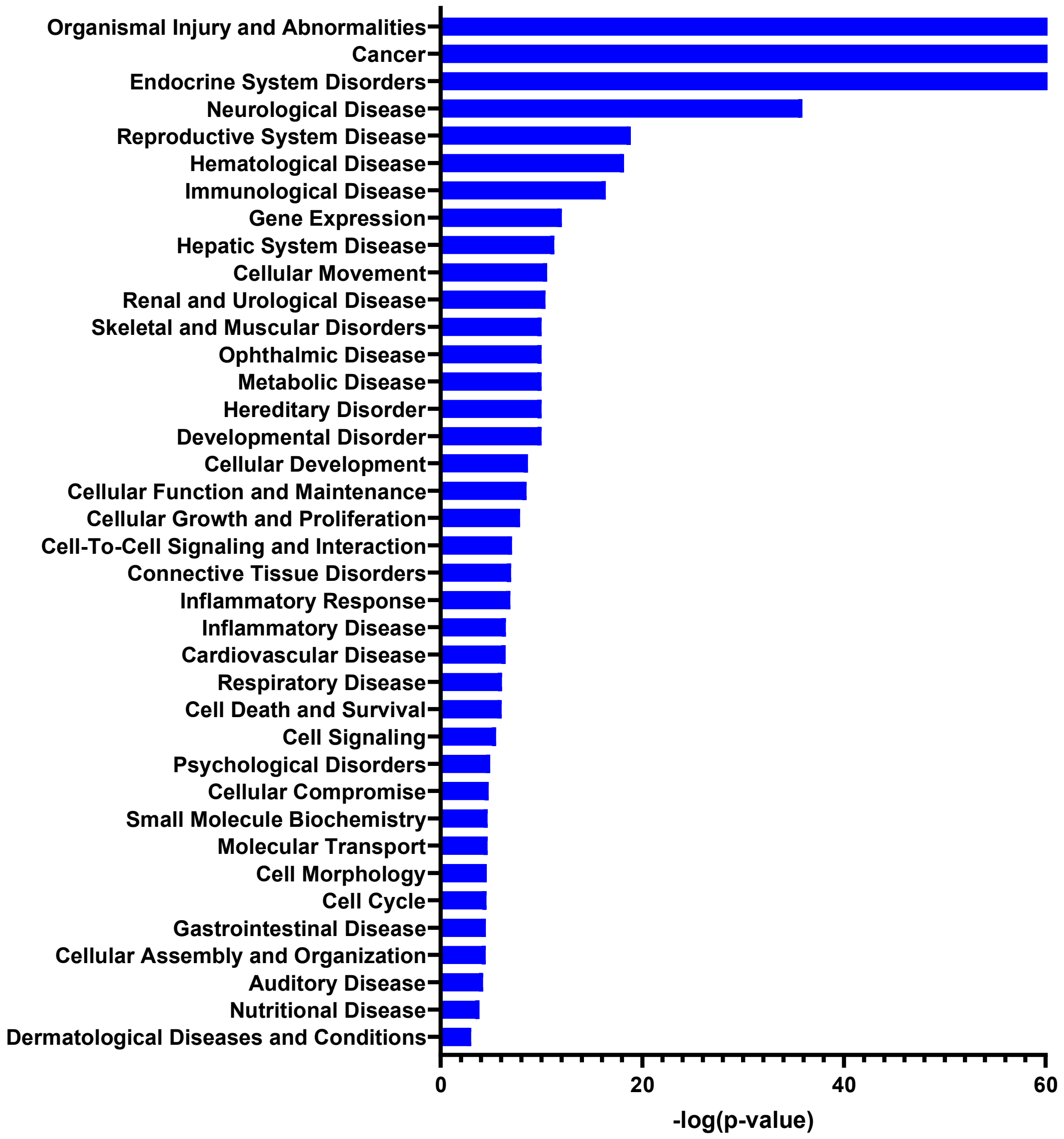

Figure S8. *Significant Molecular and Cellular Functions identified by Ingenuity Pathway Analysis (IPA) when using the downregulated DEGs between steatotic and Ex-4-treated steatotic cells.*

Table S3. Complete list of 31 overlapping DEGs between steatotic cells and Ex-4-treated steatotic cells. Genes downregulated in steatotic, relative to untreated cells (UCs), and upregulated after Ex-4 treatment are in green, while those upregulated in steatotic cells (StCs), relative to untreated cells, and downregulated after Ex-4 treatment (EX-4-TStCs) are in red

Name	Fold change between StCs and UCs	Fold change between Ex-4-TStCs and StCs
TNFRSF6B	-508.49	437.85
TBC1D3G	-137.85	62.46
ZBED6	-107.46	102.73
PSG8	-58.58	25.94
HTR6	-14.3	55.24
AL157392.5	-6.68	87.6
PCED1B	-5.14	9
AC021066.1	-4.91	15.23
UBE2F-SCLY	-4.88	20.41
NKX2-1	-4.85	17.32
NAPSA	-4.33	8.16
AC010547.4	-4.19	148.31
COL11A2	-4.06	5.05
COL13A1	-3.73	7.69
AQP1	-3.22	65.97
RGS4	-2.83	9.94
CCNG2	-2.81	2.75
PNMA2	-2.77	12.41
TMC1	-2.03	7.12
**CES1**	**2.04**	**-5.11**
**H4C3**	**2.13**	**-3.35**
**SLC16A6**	**3.78**	**-3.85**
**AC068631.3**	**4.06**	**-5.29**
**TBX15**	**4.81**	**-8.26**
**KRT37**	**5.03**	**-11.08**
**SPINK1**	**6.72**	**-10.55**
**AC008403.1**	**11.15**	**-10.64**
**TEX28**	**43.07**	**-45.88**
**AL451062.4**	**71.9**	**-315.92**
**ARL2-SNX15**	**175.41**	**-628.52**
**AC078927.1**	**233.03**	**-17.99**
